# Unraveling Hydride‐Driven Multiphasic Reduction Toward Tunable Germanium Structures for Lithium‐Ion Batteries

**DOI:** 10.1002/advs.74278

**Published:** 2026-02-05

**Authors:** Gijung Lee, Jieun Kang, Jin Yong Kwon, Woori Bae, Noh‐Moon Lee, Byeongho Park, Yujin Park, Changwan Sun, Jihee Yoon, Hyungmin Park, Bonjae Koo, Jin Woo Yi, Jaegeon Ryu

**Affiliations:** ^1^ Department of Chemistry and Biomolecular Engineering Sogang University Seoul Republic of Korea; ^2^ School of Chemical and Biomolecular Engineering Georgia Institute of Technology Atlanta Georgia USA; ^3^ Korea Conformity Laboratories (KCL) Jeonnam CCU (Carbon Capture and Utilization) Center Yeosu Republic of Korea; ^4^ School of Chemistry and Energy Sungshin Women's University Seoul Republic of Korea; ^5^ Composites & Convergence Materials Research Division Korea Institute of Materials Science (KIMS) Changwon Republic of Korea

**Keywords:** bulk germanium anodes, hydride‐mediated reduction, lithium‐ion batteries, multiphasic reduction

## Abstract

Germanium (Ge) stands out as a promising anode due to its high theoretical capacity combined with intrinsically superior ionic and electronic conductivities. Nevertheless, the high cost and pronounced volume expansion upon lithiation pose significant challenges for its practical implementation. Herein, sodium hydride (NaH)‐driven multiphasic reduction is introduced to synthesize micrometre Ge with a tailored porous and hybrid nanocrystalline‐amorphous structure, which uniquely emerges under off‐stoichiometric reduction conditions. By elucidating the underlying multiphase reaction pathways, this structural evolution can be attributed to the dual role of NaH decomposition, where hydrogen regulates porosity and crystallinity while metallic Na acts as the primary reductant for germanium dioxide. This synthesized Ge exhibits outstanding reversibility and an exceptionally cycling stability even at high current density compared to commercial Ge microparticles, while also preserving the electrode integrity throughout cycling. This study offers mechanistic insights into extending NaH‐driven reduction beyond GeO_2_ to other metal oxides, paving the way for the development of high‐capacity anodes.

## Graphical abstract

The sodium hydride (NaH)‐mediated reduction of germanium dioxide yields distinct Ge structures depending on the amount of reductant, revealing the dual roles of NaH in the reduction pathways. The synthesized Ge under off‐stoichiometric conditions exhibits a porous morphology and reduced crystallinity, which effectively mitigates volume expansion during cycling and leads to stable electrochemical performance compared to commercial Ge.

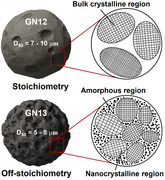

## 1 Introduction

With the ongoing shift toward electrification and sustainable mobility, the demand for lithium‐ion batteries (LIBs) has surged as interest in advanced energy storage systems continues to grow. As the limited energy density of conventional graphite anode (372 mAh g^−1^) constrains LIB performance, considerable efforts have focused on alternative high lithium (Li)‐stoichiometric anodes as represented by group IV elements (e.g., Si and Ge) [1, 2, 3]. Although Si offers the highest specific capacity, its practical use is severely limited by drastic volume expansion (>300%), which triggers mechanical failure, particle pulverization, and electrical disconnection [4, 5]. Also, the intrinsically poor Li^+^ diffusivity and low electronic conductivity of Si cannot be fully resolved, thereby limiting its widespread applications. As an alternative, Ge has drawn attention for its favorable combination of high gravimetric (1396 mAh g^−1^) Li storage capacity for Li_3.75_Ge, along with fast ion and electron transport properties compared to Si. However, the practical implementation of Ge anodes has been hindered by high material cost and structural instability arising from large volume changes [6, 7].

Driving their stress‐mitigating structure in a scalable manner has been primarily achieved via the thermochemical reductions of cost‐effective oxide‐based precursor (e.g., germanium dioxide (GeO_2_)), such as wet‐chemical reduction [8], thermal reduction [9, 10, 11], sputtering deposition [12], colloidal synthesis [13], and molten salt synthesis [14]. These methods enable a facile tailoring of the structural configuration of particles, particularly pore engineering. This approach effectively accommodates the substantial volumetric changes associated with cycling, while the internal pore network serves as a mechanical buffer that suppresses pulverization [15, 16]. Moreover, the interconnected porous framework facilitates electrolyte infiltration and enhances Li^+^ accessibility to active sites, further improving electrochemical performances [17]. Besides, tailored crystallinity of Ge impacts the structural resilience through the robust control of the stress evolution behavior and lithiation dynamics [18]. Unlike crystalline particles, amorphous structures generally enable isotropic stress dissipation, thereby facilitating uniform strain accommodation and providing sufficient free volume to mitigate structural disruption [19]. Amorphous materials also exhibit relatively low energy barriers for Li^+^ diffusion compared to their crystalline counterparts. Accordingly, amorphous structures are generally favored for enhanced stability and capacity, as their homogeneous volume expansion during Li^+^ insertion mitigates mechanical degradation [20]. Additionally, the presence of nanocrystallites embedded in an amorphous matrix further facilitates ion transport through hopping mechanisms, leading to improved electrical conductivity [21]. In addition to porosity and crystallinity, particle size is another key structural parameter. While nanoscale Ge promotes parasitic side reactions due to its large surface area, micrometre Ge exhibits reduced surface reactivity, higher tap density, potentially enabling the energy‐dense electrode formulation [22]. Ultimately, such structural characteristics contribute significantly to the enhancement of electrochemical performance and mechanical robustness.

Although individual structural parameters can offer certain advantages, stable electrochemical performance cannot be achieved by manipulating only one structural feature. In practice, conventional reduction methods of GeO_2_ are limited in simultaneously tuning size, morphology, and crystal structure [23]. The integration of these architectural characteristics can generate synergistic effects, suppressing the detrimental volume expansion while enhancing interfacial stability [24]. Furthermore, a detailed mechanistic understanding underlying these methods remains elusive. Without such insight, the reduction process remains largely empirical, restricting scalability and reproducibility. Especially, in sodium hydride (NaH)‐mediated reduction of metal oxides, the role of NaH is still unclear [25]. Although its decomposition likely generates multiple reactive species that may contribute differently to the reduction process, such pathways have not been systematically reported. These challenges underscore the need for systematic investigations of the underlying mechanisms to guide the development of synthetic routes capable of concurrently controlling structural parameters.

**SCHEME 1:**
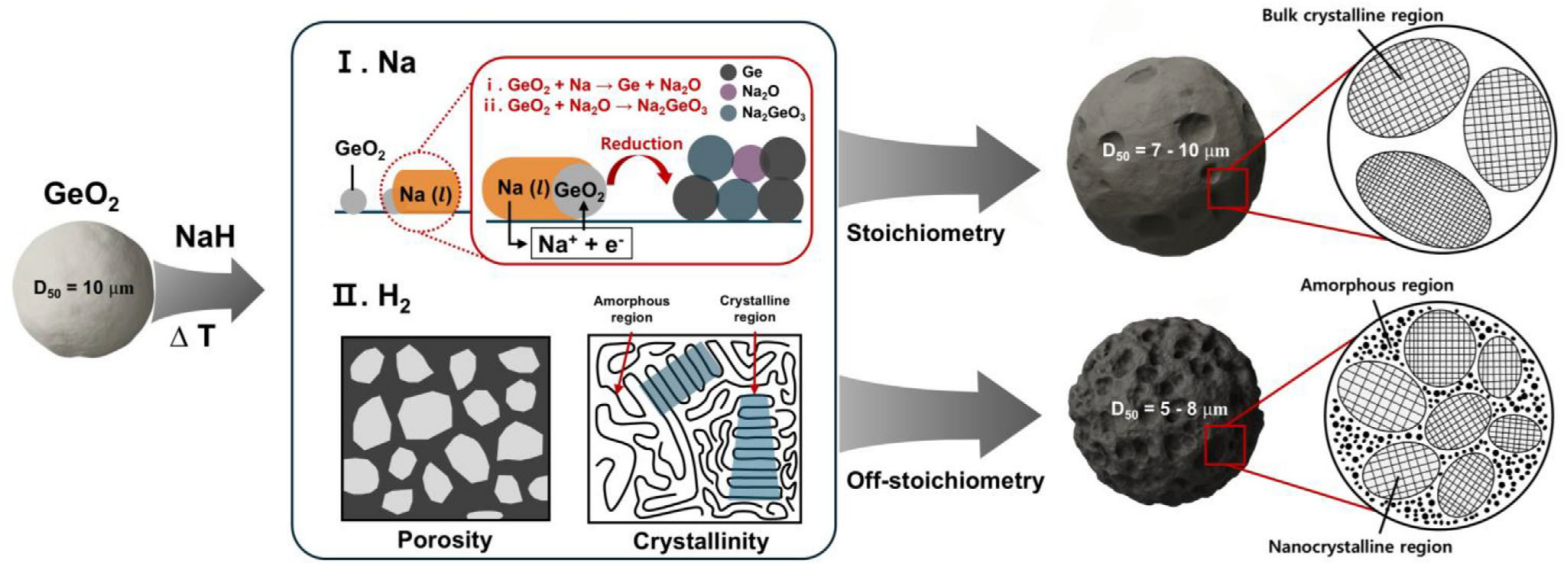
Schematic illustration of the dual role of NaH in the reduction of GeO_2_ to Ge and its influence on porosity and crystallinity.

Herein, we elucidate, for the first time, the mechanism of NaH‐mediated multiphase reduction to synthesize a porous micro‐sized Ge with a controlled crystallinity through a straightforward and facile synthetic method (Scheme 1). Upon thermal annealing of precursor‐GeO_2_ with NaH in the closed system [26], the GeO_2_ reacts with Na generated from NaH to form a bulk structure of Ge. Notably, the morphology and crystallinity of the synthesized bulk Ge were found to be strongly dependent on the amount of hydrogen vapors released from NaH used. Under strong reducing conditions with higher NaH content, enhanced hydrogen evolution promotes the formation of porous Ge with a mixed structure of nanocrystalline and amorphous phases. As a result, this structure obtained from higher NaH content effectively relieves internal electrode stress, ensuring sustained stability during long‐term battery cycling, even at high C‐rate conditions. These findings shed light on the mechanistic role of NaH in metal oxide reduction, suggesting that this strategy may apply to other metal oxides for the development of high‐capacity anodes.

## 2 Results and Discussion

**FIGURE 1:**
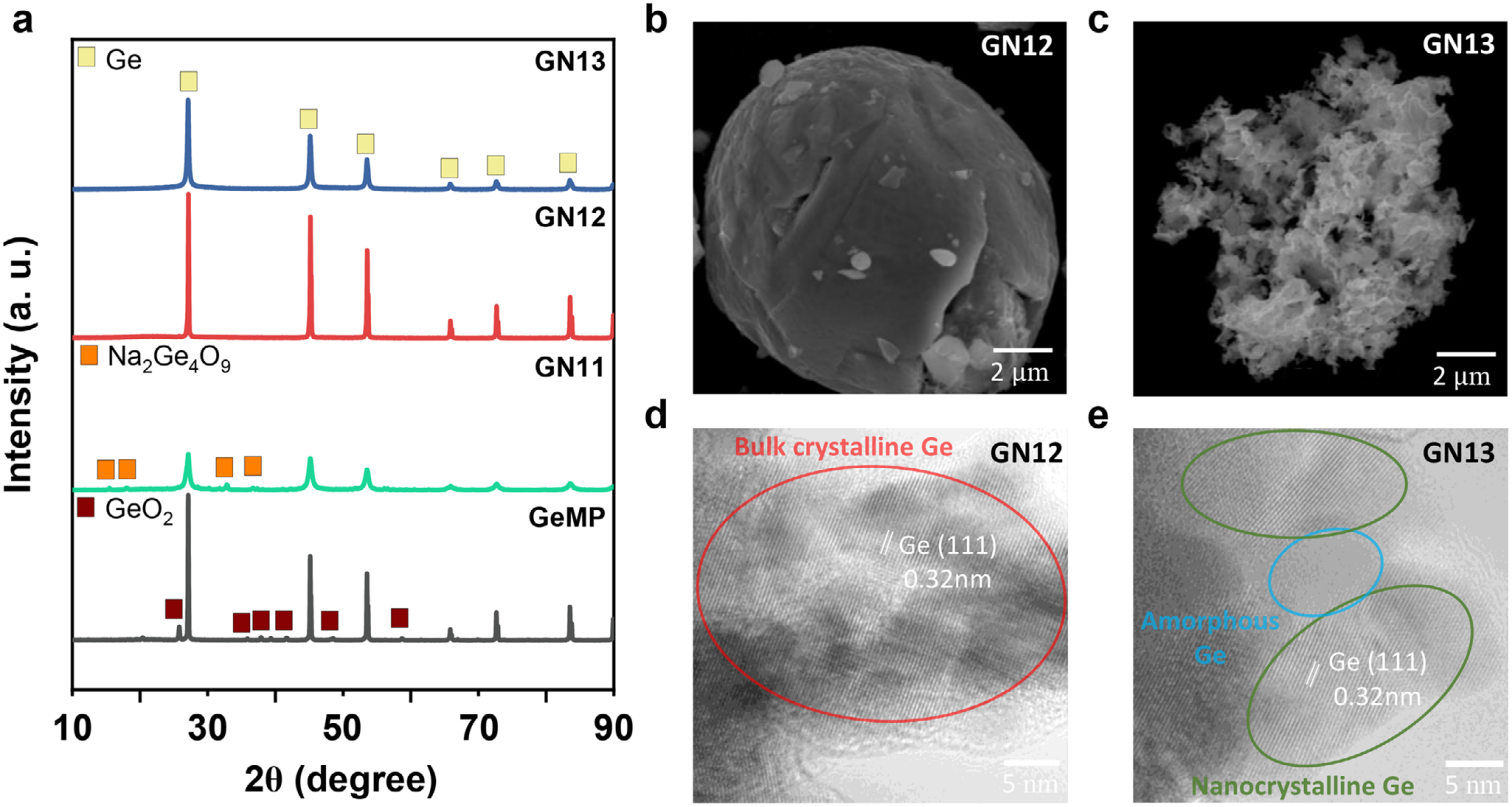
Phase and morphological characterization of Ge obtained by NaH‐mediated reduction of GeO_2_. (a) XRD patterns of GeMP, GN11, GN12, and GN13. FE‐SEM images of (b) GN12 and (c) GN13. TEM images of (d) GN12 and (e) GN13.

The Ge microstructures synthesized by NaH‐mediated reduction of bulk GeO_2_ (Figure S1) were investigated under a GeO_2_ to NaH molar ratio of 1.0 to 4.0 and are denoted GN11, GN12, GN13, and GN14 (e.g., GN12, corresponding to a 1:2 molar ratio of GeO_2_:NaH). In GN12 and GN13, the X‐ray diffraction (XRD) patterns show a distinct phase transformation from GeO_2_ to Ge, while commercial Ge microparticles (GeMP) still exhibit GeO_2_ reflections attributable to native oxide layers (Figure 1a). In contrast, GN11 reveals diffraction peaks corresponding to Ge and sodium tetragermanate (Na_2_Ge_4_O_9_). This implies that the insufficient reductant hindered the reduction of GeO_2_, yielding Ge_4_Na_2_O_9_ intermediates. The detailed mechanistic pathways underlying the NaH‐mediated reduction will be discussed in a subsequent section. Notably, GN14 is dissolved in deionized water during the washing process (Figure S2). For GN14, the use of excessive NaH results in the formation of a strongly alkaline solution during the washing process (Figure S3), arising from the hydrolysis of residual NaH to NaOH as represented by the following Equation (1):

(1) $$\mathrm{NaH}\;\left(s\right)+H_{2}O\;\left(l\right)\;\to \;\mathrm{NaOH}\;\left(\mathrm{aq}\right)+H_{2}\;\left(g\right)$$

Owing to the amphoteric nature of Ge, such an alkaline environment induces the dissolution of Ge into germanate ions and results in the loss of the product after purification [27, 28]. Accordingly, GN14 was excluded from subsequent structural characterization and electrochemical evaluation.

The as‐synthesized GN12 and GN13 exhibit distinct morphologies under different reducing environments, yet in a similar particle size of 5–10 µm (Figure S4). GN 12 retains a microspherical morphology (Figure 1b), while GN13 transforms into a highly porous framework (Figure 1c). This porous transformation can be attributed to the vigorous hydrogen evolution under stronger reducing conditions [29]. The transmission electron microscope (TEM) image further reveals distinct structural features between GN12 and GN13. The GN12 predominantly consists of bulk crystalline Ge domains with (111) lattice fringes of Ge (*d* = 0.32 nm) (Figure 1d). In contrast, GN13 comprises nanocrystalline Ge domains interspersed with amorphous regions (Figure 1e). This loss of long‐range crystallinity is driven by excessive hydrogen evolution, which drives rapid hydrogen diffusion and swift oxygen removal at the oxide‐metal interface, favoring disordered growth over lattice reorganization [30].

**FIGURE 2:**
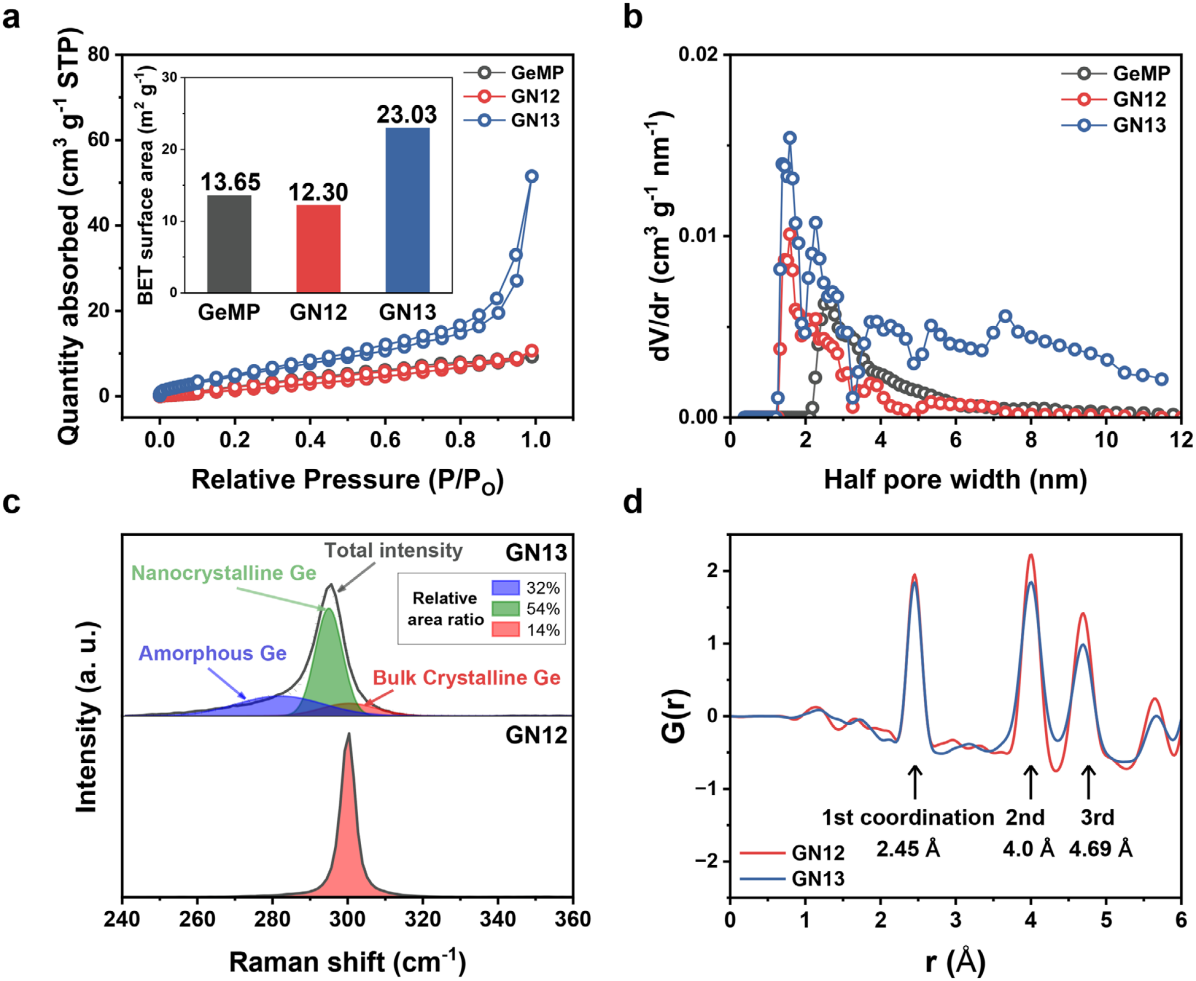
Structural characterization of GN12 and GN13 samples. (a) Nitrogen adsorption/desorption curves (Inset: BET specific surface area of samples and the differential pore size distribution plots for GeMP, GN12, and GN13. (c) Raman spectrum (Inset: relative area ratios) and (d) PDF analysis of GN12 and GN13.

To further clarify the bifunctional effect of hydrogen evolution, additional structural analyses were conducted. Porosity was characterized by the nitrogen adsorption/desorption and pore size distribution curves for the GN samples. The sorption plots of GN13 exhibit a typical type IV isotherm with a pronounced hysteresis loop of mesoporous materials, in contrast to the type II isotherm characteristic of nonporous solids observed for GeMP and GN12 (Figure 2a). Consistent with the porous framework observed in the SEM images, the GN13 reveals a markedly higher specific surface area of 23.03 m^2^ g^−1^ than GeMP and GN12. The pore size distribution further supports the structural properties of GN samples (Figure 2b). Narrow pore distributions primarily below 4 nm are observed for GeMP and GN12, indicative of limited porosity. Conversely, GN13 reveals a broad distribution of mesopores centered around 2–6 nm, with extended tails up to 10 nm. Consistent with this porous architecture, GN13 exhibits a lower tap density than GN12, yet still maintains a higher packing density than bulk Si (5 µm) with a comparable particle size (Figure S5). From these results, GN13 features a multiscale porous structure, which can effectively alleviate the severe volume expansion during lithiation. This porous architecture arises from the accelerated oxygen removal and vacancy coalescence induced by excessive hydrogen evolution, which generates internal voids through a Kirkendall effect between rapid oxygen depletion and slower Ge diffusion [29, 31].

The additional role of hydrogen in controlling crystallinity was examined by Raman spectra (Figure 2c). Specifically, excessive hydrogen evolution accelerates hydrogen diffusion and facilitates rapid oxygen removal at the oxide‐metal interface, thereby favoring disordered lattice growth and reduced crystallinity [30]. GN12 shows a sharp and intense peak at 300 cm^−1^, characteristic of bulk crystalline Ge. In comparison, GN13 presents a broadened spectrum deconvoluted into approximately 32% amorphous, 54% nanocrystalline, and 14% bulk crystalline Ge, indicating a substantially higher fraction of disordered domains [32]. To gain deeper insight into this structural disruption, pair distribution function (PDF) analysis was subsequently carried out (Figure 2d). The PDF represents the probability of locating atomic pairs at a given distance. This analysis is used to investigate local atomic structure and disorder in both amorphous and nanocrystalline materials [33]. For GN12 and GN13, the coordination distances of the first, second, and third are 2.45, 4.0, and 4.69 Å, respectively. However, the second and third peaks of GN13 are noticeably broader than those of GN12, indicating that GN13 retains a certain degree of short‐range order but shows pronounced structural disorder [34, 35]. Taken together, these results demonstrate that GN13 features a hybrid amorphous‐nanocrystalline structure that buffers volume changes in Li^+^ uptake and enhances ion transport, enabling stable interfacial contact and fast charge–discharge kinetics.

As mentioned earlier, although the amount of NaH clearly governs the characteristics of the NaH‐mediated GeO_2_ reduction, the corresponding reduction mechanism remains insufficiently elucidated. Here, we put forward a multi‐pathway reduction mechanism as below that can account for the complex reaction behavior observed in NaH‐mediated GeO_2_ reduction (Equations (2), (3), (4)), in which GN12 was revealed as the product of a stoichiometric reaction.

(2) $$4\;\mathrm{NaH}\;\left(s\right)\to \;4\;\mathrm{Na}\;\left(l\right)+2\;H_{2}\;\left(g\right)$$

(3) $$\mathrm{Ge}O_{2\;}\left(s\right)+4\;\mathrm{Na}\;\left(l\right)\to \;\mathrm{Ge}\;\left(s\right)+2\;Na_{2}O\;\left(s\right)\;$$

(4) $$\mathrm{Ge}O_{2}\left(s\right)+\;Na_{2}O\;\left(s\right)\to \;Na_{2}\mathrm{Ge}O_{3}\;\left(s\right)\;$$

(5) $$\begin{array}{ccc} & & \mathrm{Overall}\;\mathrm{reaction}:\\ & & \;2\;\mathrm{Ge}O_{2}+4\;\mathrm{NaH}\;\to \;\mathrm{Ge}+Na_{2}O+Na_{2}\mathrm{Ge}O_{3}+2\;H_{2} \end{array}$$

**FIGURE 3:**
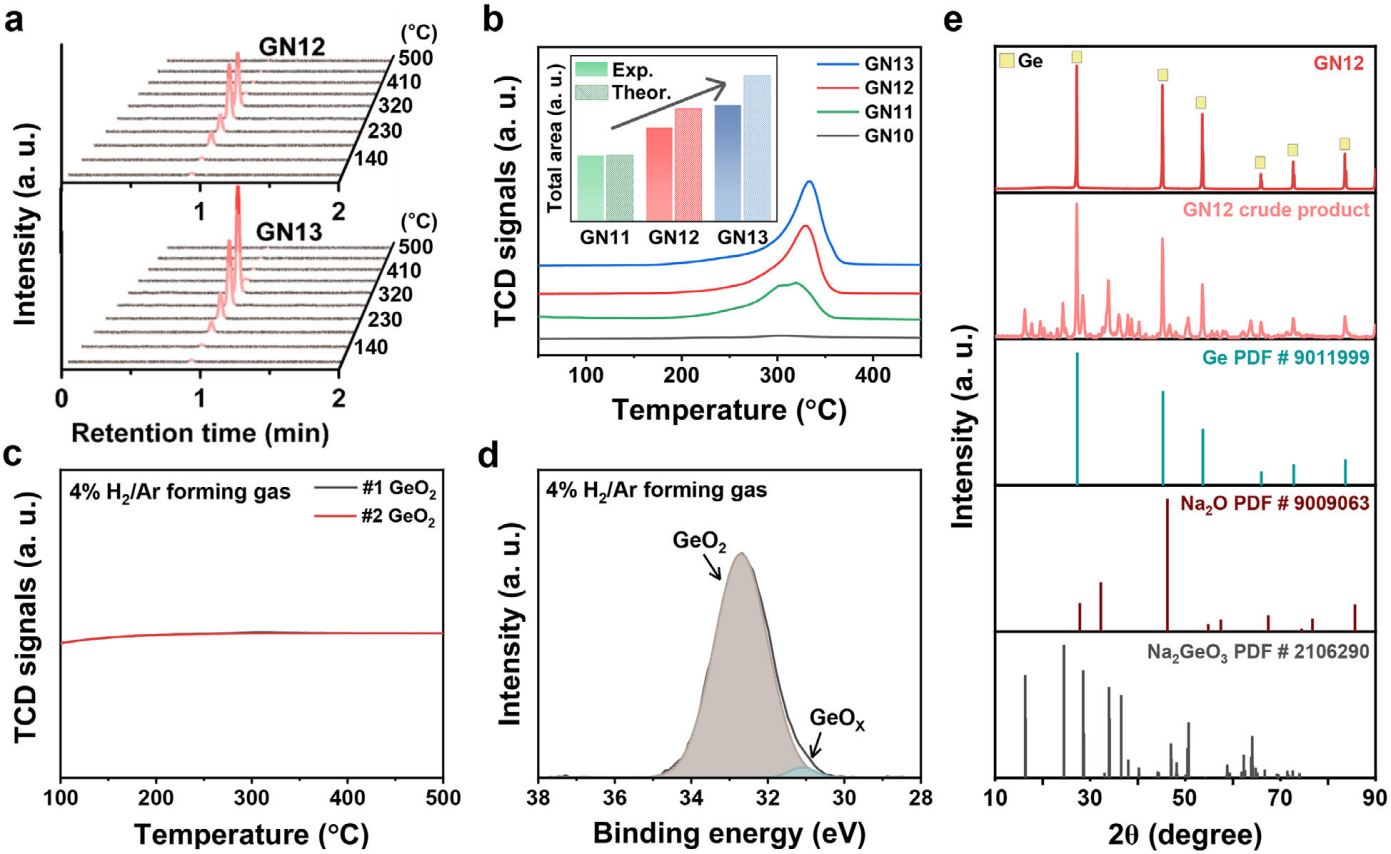
Investigation on the mechanism of NaH‐mediated reduction of GeO_2_. (a) Temperature‐dependent GC profiles of GN12 and GN13. (b) Variation of TCD signals of GN10, GN11, GN12, and GN13 with temperature under Ar flow. (c) Variation of TCD signals of GeO_2_ with temperature under 4% H_2_/Ar forming gas. (d) Ge 3d XPS spectrum of GeO_2_ reduced in product.4% H_2_/Ar forming gas. (e) XRD patterns of GN12 crude.

First, we investigated the decomposition of NaH with the associated hydrogen evolution (Equation 2). The evolution of gaseous products from the GeO_2_‐NaH mixture was examined by gas chromatography (GC) during heating up to 500°C. For GN12 and GN13 mixtures, the GC profiles consistently showed the evolution of hydrogen as the sole gaseous species (Figure 3a). Likewise, the GN11 mixture also released only hydrogen, whereas the NaH‐free system (GN10 mixture) showed no detectable gas evolution (Figure S6). This indicates that no other volatile products were generated under the applied conditions. Also, temperature programmed desorption (TPD) analysis was performed to quantify the hydrogen evolution associated with NaH decomposition (Figure 3b). For the GN10 mixture, the absence of hydrogen release under Ar flow led to no detectable variation in the thermal conductivity detector (TCD) signal. In contrast, GN11, GN12, and GN13 mixtures exhibited noticeable changes in the TCD signal with varying peak areas, implying differences in the amount of hydrogen released. Integration of the TCD peaks enabled quantification of the hydrogen released, and comparison with the theoretical values confirmed that the amount of hydrogen increases progressively from GN11 to GN13 mixtures in agreement with the expected stoichiometry. Therefore, the decomposition of NaH determines the extent of hydrogen evolution, which directly influences the structural characteristics of the reduced products.

Next, Na (*l*) generated from NaH decomposition acts as the primary reductant for GeO_2_. Primarily, to test the possibility of GeO_2_ reduction by hydrogen at 500°C, TCD signals of GeO_2_ were monitored under an 4% H_2_/Ar (forming gas) environment (Figure 3c). Despite repeated measurement, no detectable shift was observed, suggesting that hydrogen uptake did not occur and thus no reduction was detected by the TPD analysis. In addition, an attempt was made to reduce GeO_2_ with forming gas under the same conditions used for GN sample synthesis. XRD patterns revealed that the diffraction peaks of the treated GeO_2_ remain unchanged from those of pristine GeO_2_ (Figure S7). Since GeO_2_ could undergo partial reduction at the surface, X‐ray photoelectron spectroscopy (XPS) analysis was carried out (Figure 3d). The Ge 3d spectra from the treated GeO_2_ displayed a dominant GeO_2_ peak, along with partially reduced GeO*_x_* peaks [36]. These results indicate that GeO_2_ is not reduced by hydrogen under 500°C, demonstrating that hydrogen does not serve as the primary reductant in the NaH‐mediated reduction [37].

The Ellingham diagram that delineates the temperature‐dependent stability of oxides provides a thermodynamic basis for predicting the feasibility of reduction reactions. According to the Gibbs free energy differences of the respective reactions, metallic Na possesses sufficient reducing power to convert GeO_2_ into Ge, indicating the strong driving force of Na for oxide reduction [38, 39]. Thus, the NaH‐mediated reaction proceeds to produce Ge and sodium oxide (Na_2_O), as shown in Equation (3). To confirm the formation of Na_2_O, the crude product obtained after the NaH‐mediated reaction was analyzed by XRD before washing (Figure S8a). Since the peak intensity of Na_2_O is much weaker than that of other metallic peaks, the XRD pattern was enlarged to clearly resolve the Na_2_O reflections. Among the diffraction peaks of Na_2_O, the most intense (220) reflection at 46° was clearly observed in the crude product of GN12. As GC analysis revealed the evolution of H_2_ but no detectable O_2_, the formation of Na_2_O is ascribed to the reduction of GeO_2_ by Na, thereby confirming that Na is the predominant reductant.

In the XRD pattern of the GN12 crude product, intense reflections are observed at approximately 16°, 24°, and 28°, assigned to sodium germanate (Na_2_GeO_3_) (Figure S8b), suggesting that a new reaction pathway occurs in the NaH‐mediated reduction, as presented in Equation (4). As a result, the crude product of GN12 mainly consists of Ge, Na_2_O, and Na_2_GeO_3_, while the minor reflections are likely attributed to nonstoichiometric Na*_x_* Ge*_y_* O*_z_* phases or minor Na‐containing byproducts (Figure 3e). These secondary phases are readily removed during the washing process using deionized water, resulting in the formation of pure Ge in the final GN12 sample. Notably, unlike conventional GeO_2_ reduction methods that require acidic treatments for byproduct removal [40, 41], this process enables purification via simple water washing, underscoring the environmental sustainability of the process. Furthermore, GN11 and GN13 crude products were also examined. In contrast to GN12, the crude product of GN11 exhibited additional peaks near 20° and 26°, corresponding to GeO_2_ (Figure S9a). After washing, the product was found to contain Ge together with the intermediate Na_2_Ge_4_O_9_, which is sparingly soluble in water. This indicates that the amount of NaH was insufficient to completely reduce GeO_2_, as the NaH/GeO_2_ ratio was lower than the stoichiometric requirement. In GN13 crude product, the use of excess NaH promotes the formation of additional byproducts, which results in overlapping reflections and complicates the unambiguous identification of individual peaks (Figure S9b). Nevertheless, the key point is that such phases dissolve upon washing, resulting in phase‐pure Ge. Consequently, GN12 follows the ideal stoichiometric pathway, while GN11 is limited by insufficient reductant, and GN13 processes under reductant excess. The overall reaction of GN12 is represented in Equation (5).

The GN samples were comparatively examined in coin‐type half‐cell tests to assess their Li storage behavior. The cyclic voltammetry (CV) curve of GN12 and GN13 electrodes exhibits three major cathodic peaks at 0.36 V (peak 1), 0.15 V (peak 2), and 0.015 V (peak 3) (Figure S10) [42]. In the GN13 electrode, the amorphous domains play a decisive role in the electrochemical response. This structural characteristic facilitates more frequent amorphous‐amorphous (*a*‐*a*) transitions during delithiation, which results in the more pronounced intensity of peak 4 compared to GN12 [43]. Furthermore, peak 5 observed in the GN12 electrode, which corresponds to the crystalline‐amorphous (*c*‐*a*) transition, shifts to a lower potential of 0.59 V in the GN13 electrode. Such a shift indicates the reduced kinetic barrier associated with the breakdown of the closely packed crystalline lattice into a disordered amorphous Li*_x_* Ge phase, ultimately facilitating a more efficient and reversible lithiation/delitiation process [43]. Moreover, galvanostatic intermittent titration technique (GITT) measurements confirmed that the GN13 electrode possesses a higher Li^+^ diffusivity compared to the GN12 electrode (Figure S11). Combined with the CV analysis, this indicates that the coexistence of amorphous and nanocrystalline domains in the GN13 electrode enhanced redox kinetics.

**FIGURE 4:**
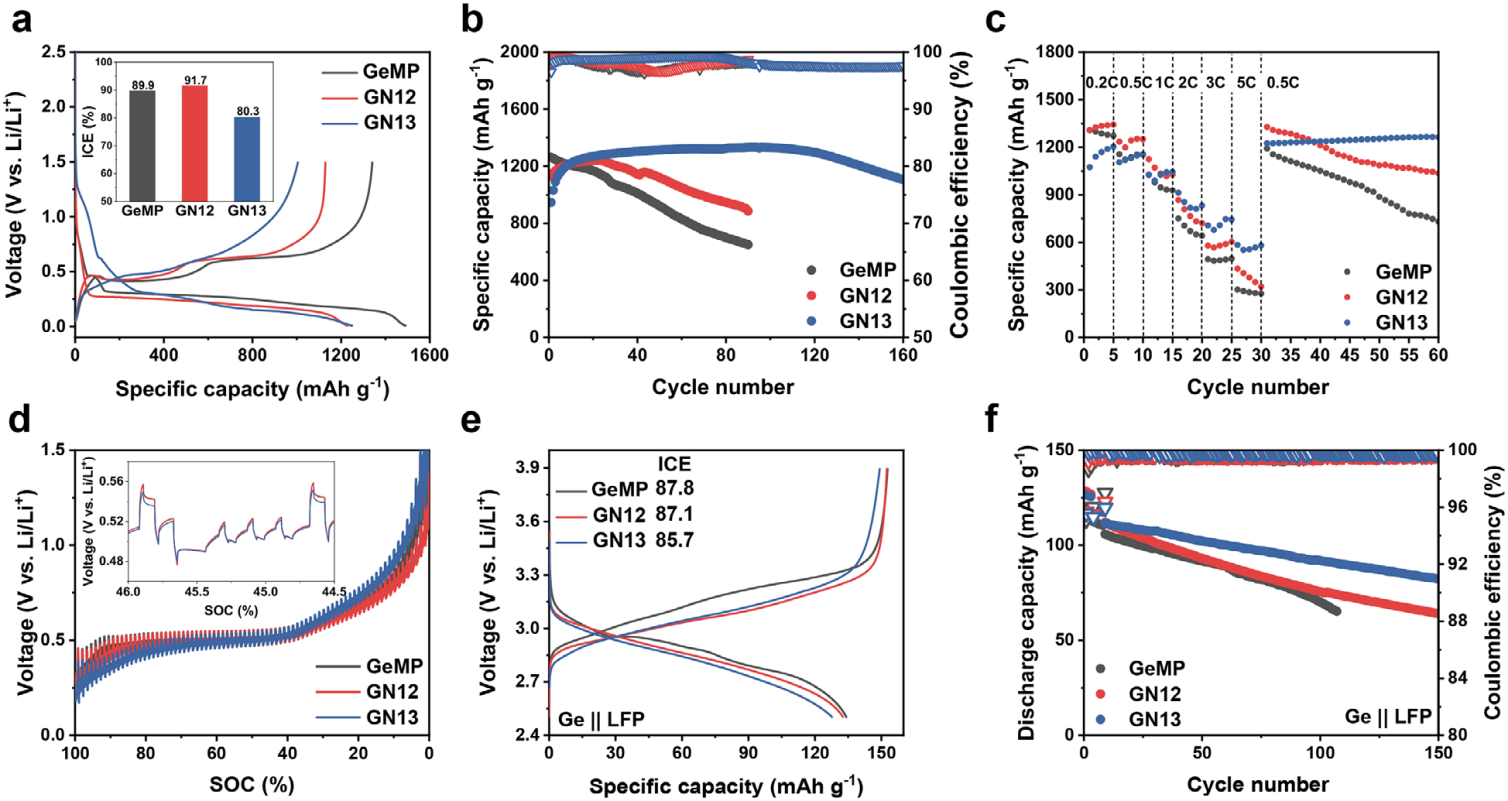
Electrochemical performance with half‐cells and full‐cells of GeMP, GN12, and GN13 electrodes. (a) Galvanostatic charge–discharge profiles in half‐cells (Inset: initial columbic efficiency) of GeMP, GN12, and GN13 electrodes. (b) Long‐term cycling performance and corresponding CEs of the GeMP, GN12, and GN13 half‐cells at 0.2C (1C = 1.2 A g^−1^). (c). Rate capability of GeMP, GN12, and GN13 half‐cells. (d) USABC dynamics stress test of GeMP, GN12, and GN13 half‐cells. (e) Galvanostatic charge‐discharge profiles of the GeMP || LFP, GN12 || LFP, and GN13 || LFP full cells. (f) Long‐term cycling performance and corresponding CEs of the GeMP || LFP, GN12 || LFP, and GN13 || LFP full cells at 0.33C (1C = 135 mA g^−1^).

The electrochemical performance was evaluated in half‐cell configurations. The GN13 electrode exhibited a slightly lower initial Coulombic efficiency (ICE) compared to GN12 and GeMP electrodes during the formation cycle at 0.05C (1C = 1.2 A g^−1^), which can be ascribed to its porous structure (Figure 4a). Nevertheless, this structural feature provides additional active sites and facilitates electrolyte penetration, ultimately contributing to enhanced long‐term electrochemical performance. Consistently, long‐term cycling stability evaluated at various C‐rates revealed that the GN13 electrode maintained high coulombic efficiency (CE) and exceptional capacity retention at 0.2C and 0.5C (Figure 4b **;** Figure S12, and Table S1). In contrast, the GN11 electrode containing residual Na_2_Ge_4_O_9_ shows poor cycling behavior with low ICE, slow CE recovery, and ultimately low capacity retention, primarily due to the irreversible reactions associated with the Na_2_Ge_4_O_9_ phase (Figure S13). Furthermore, rate performance of GN electrodes at C‐rate of 0.2C to 5C revealed that the specific capacities of GN13 electrode at 3C and 5C were 716 and 568 mAh g^−1^, significantly outperforming the GN12 and GeMP electrodes (Figure 4c). Such superior long‐term stability and rate capability of the GN13 electrode are attributed to its robust structural stability arising from porous architecture combined with amorphous and nanocrystalline domains, which together mitigate volume variation and ensure efficient Li^+^ transport during repeated cycling even under elevated C‐rates. In particular, the dynamic performance can be assessed by the United States Advanced Battery Consortium (USABC) dynamic stress test (DST) protocol, which measures polarization under successive regenerative current pulses and rapid C‐rate variations (Figure S14) [44]. Compared with GN12 and GeMP electrodes, the GN13 electrode exhibits lower polarization and a more stable voltage response (Figure 4d). Therefore, the distinctive porous and hybrid amorphous‐nanocrystalline structure of GN13 validates its superior electrochemical performance.

The suppression of drastic volume expansion during cycling is critical for preserving electrode integrity and stabilizing both bulk and interfacial structure. In this regard, the structural and interfacial characteristics analysis of GN samples further reveals how intrinsic structural properties affect performance. Initially, the swelling behavior was monitored even after extended cycling (Figure S15). The GN13 electrode showed significantly suppressed swelling of 48.1%, less than half that of the GeMP and GN12 electrodes. Top‐view SEM images further corroborate the swelling behavior of GN sample electrodes, where the GN13 electrode displays significantly fewer cracks and maintains a more uniform surface than GeMP and GN12 electrodes (Figure S16). The suppression of cracking was evident at both electrode and particle levels. By buffering mechanical stress and mitigating volume expansion, GN13 effectively suppressed particle pulverization and excessive solid electrolyte interphase (SEI) growth, unlike GN12 (Figure S17). To further probe the surface morphology of GN12 and GN13, atomic force microscopy (AFM) was performed (Figure S18). With a scan area (8  ×  8 µm) comparable to particle size, the uneven height distribution of GN12 reflects surface roughening at the particle level that accelerates degradation of the electrode network, whereas GN13 showed a relatively smooth surface due to a stress‐dispersing structure.

In addition to the mechanical robustness of the bulk structure, the electrode‐electrolyte interface was investigated by XPS (Figure S19). In the C 1s spectra, the GN12 electrode shows a higher intensity of C‐C bonds after cycling compared to the GN13 electrode, indicating the accumulation of organic SEI components originating from persistent electrolyte decomposition [45]. Moreover, O 1s spectra revealed that the GN13 electrode exhibits a higher contribution of Li_2_O species in the etched spectra compared with the GN12 electrode, suggesting the formation of a uniformly organized inorganic SEI layer facilitated by uniform Li^+^ transport, and the GN13 electrode effectively suppresses further decomposition of SEI during cycling [46]. Electrochemical impedance spectroscopy (EIS) was further utilized to probe resistance variations from the interface to the bulk of the electrode during cycling (Figure S20a,b). The Nyquist plots reveal that the GN12 electrode exhibits a continuous rise in impedance with prolonged cycling, whereas the GN13 electrode maintains relatively lower impedance evolution. To gain deeper insights, the distribution of relaxation time (DRT) that distinguishes bulk resistance (R_bulk_), SEI resistance (R_SEI_), and charge transfer resistance (R_ct_) was performed (Figure S20c,d). The GN12 electrode shows a marked rise in R_bulk_ due to electrolyte depletion and microcrack formation within the electrode, both of which hinder ionic transport and increase contact resistance [47]. In contrast, the GN13 electrode exhibits a much smaller rise in R_bulk_, indicating a more stable ionic conduction network and reduced structural degradation. Furthermore, GN13 electrode exhibits small increases in R_SEI_ and R_ct_ than GN12 electrode, further confirming that suppressed volume expansion facilitates the formation of stable SEI and improved charge transfer [48].

Based on the mechanical robustness and interfacial stability arising from the structural features of GN13 electrode, full‐cell performance was further evaluated by pairing the GN anodes with the LiFePO_4_ (LFP) cathode in a coin cell with an N/P ratio of 1.1 (Figure 4e and Figure S21) [49]. Although the ICE of GN13 is lower, the GN13 full‐cell exhibits remarkable cycling stability at 0.33C (1C = 135 mA g^−1^) (Figure 4f). The GN13 full‐cell exhibits more rapid saturation of CE and maintains higher values than GN12 and GeMP, while also delivering superior capacity retention for 150 cycles. Furthermore, the GN13 full‐cell sustains higher discharge capacities from 0.1C to 2C compared to GN12 and GeMP (Figure S22). In addition, pouch‐cell assembled with identical GN13/C anode and LFP cathode exhibited cycling behavior consistent with that of 2032 coin cells, confirming stable performance upon scaling (Figure S23). As a result, such enhanced cycling performance of the GN13 full‐cell can be attributed to the structural integrity and interfacial stability afforded by the distinctive architecture of GN13.

Despite the demonstrated electrochemical stability in both half‐ and full‐cells, the relationship between electrode porosity and cell energy density remains unclear, as highly porous structures can accommodate volume changes but often compromise practical energy density. Accordingly, optimizing electrode porosity has recently gained increasing attention, particularly for alloying‐type anodes. Here, the porosity of the materials and electrodes was approximately estimated (Table S2) [50]. GeMP, GN12, and GN13 exhibited high anode porosities (P_anode_) of 81.1, 81.8, and 83.0%, respectively, far exceeding commercial requirements. Considering the negligible intraparticle porosity (P_intra_) of GeMP and the tap density results (Figure S5), GN12 is dominated by interparticle porosity (P_inter_), whereas GN13 contains substantial intraparticle porosity, consistent with morphological observations. Notably, sufficient closed pores, as in GN13, can mitigate losses in gravimetric energy density. Therefore, these electrodes should be further densified via high‐solid‐content slurries and calendaring to enhance volumetric performance. As a result, all three electrodes were compacted to reduce P_inter_ and P_anode_, achieving a high volumetric capacity (>790 mAh cm^−^^3^), well above that of practical graphite anodes (≈550 mAh cm^−^^3^). Overall, porous active materials should be carefully engineered at both the material and electrode levels, and pore architectures that maximize closed pores while minimizing exposed open pores should be prioritized to suppress electrolyte decomposition and improve cell‐level energy density.

## 3 Conclusions

This study presents a systematic strategy for engineering high‐performance Ge‐based anodes by leveraging a NaH‐mediated reduction to finely regulate the structural characteristics of micro‐sized Ge particles. Through an off‐stoichiometric reduction of GeO_2_ with NaH, we synthesized GN13 with a hybrid nanocrystalline‐amorphous domains within a porous framework, unlike GN12. Such structural differences originate from the underlying reduction pathway, where analysis of three multiphase routes revealed the dual role of NaH decomposition: metallic Na as the primary reductant for GeO_2_ and hydrogen as a regulator of porosity and crystallinity. Enhanced hydrogen in the reduction process led to the unique structure of GN13, imparting superior mechanical robustness to suppress volume expansion and preserving electrode integrity during cycling while simultaneously facilitating Li^+^ transport. Consequently, despite its microscale dimensions, the GN13 electrode demonstrated markedly improved rate performance and stable long‐term cycling compared to GeMP and GN12 electrodes, validated consistently in both half‐cell and full‐cell configurations. In essence, NaH‐mediated reduction may be extended to other metal oxides, potentially offering a versatile pathway for advancing high Li‐stochiometric anodes.

## Conflicts of Interest

The authors declare no conflicts of interest.

## Supporting information




**Supporting File**: advs74278‐sup‐0001‐SuppMat.docx.

## Data Availability

The data that support the findings of this study are available on request from the corresponding author upon reasonable request.
