# Supplementary material for: Unraveling Hydride‐Driven Multiphasic Reduction Toward Tunable Germanium Structures for Lithium‐Ion Batteries
Source: Adv Sci (Weinh). 2026 Feb 5;13(22):e74278. doi: 10.1002/advs.74278 (PMC13088350; doi:10.1002/advs.74278)
Supplement: Supplementary file 1 — Supporting File: advs74278‐sup‐0001‐SuppMat.docx. [file ADVS-13-e74278-s001.docx]

Supporting Information

Unraveling Hydride-Driven Multiphasic Reduction Toward Tunable Germanium Structures for Lithium-Ion Batteries

*Gijung Lee, Jieun Kang, Jin Yong Kwon, Woori Bae, Noh-Moon Lee, Byeongho Park, Yujin Park, Changwan Sun, Jihee Yoon, Hyungmin Park, Bonjae Koo, Jin Woo Yi^*^, Jaegeon Ryu^*^*

G. Lee, J. Y. Kwon, Y. Park, Prof. J. Ryu

Department of Chemistry and Biomolecular Engineering, Sogang University, Seoul 04107, Republic of Korea
E-mail: jryu@sogang.ac.kr

Dr. J. Kang
School of Chemical and Biomolecular Engineering, Georgia Institute of Technology, Atlanta, Georgia 30332, United States

W. Bae, Dr. H. Park

Korea Conformity Laboratories (KCL), Jeonnam CCU (Carbon Capture and Utilization) Center, Yeosu 59631, Republic of Korea

N.-M. Lee, Prof. B. Koo

School of Chemistry and Energy, Sungshin Women's University, Seoul 01133, Republic of Korea

B. Park, C. Sun, J. Yoon, Dr. J. W. Yi

Composites & Convergence Materials Research Division, Korea Institute of Materials Science (KIMS), Changwon 51508, Republic of Korea

E-mail: yjw0628@kims.re.kr

**Experimental Section**

*Materials:* Germanium dioxide (GeO_2_) powder (99.999%) was purchased from Germanium Corporation of America. Ge powder (-100 mesh, 99.999%) was purchased from Alfa Aesar. Sodium hydride (NaH) was purchased from Sigma-Aldrich. Poly (acrylic acid) (PAA, Mn = 450k), Carboxymethyl cellulose (CMC), Super-P were purchased from Polysciences, Sigma-Aldrich, and MTI Korea, respectively. Lithium metal (300 µm) was purchased from Honjo Chemical. The LFP electrode was purchased from MTI Korea. All the chemicals were used without further purification.

*Synthesis of GN samples:* GeO_2_ and NaH were uniformly mixed using mortar in a glovebox at a specific molar ratio (e.g., GN12 corresponds to a GeO_2_:NaH molar ratio of 1:2). This mixture was transferred to a stainless-steel reactor in an Ar atmosphere. Next, this reactor was placed in a tube furnace and heated to 500 ℃ for 3h. After the completion of the reaction, the resulting powder was treated in deionized water under mild stirring for 5h to remove the by-product. The GN product was collected by filtration and purified by deionized water. Finally, the filtered products were obtained by drying in a vacuum at 80 ℃ for 8h. GN11, GN13, and GN14 were synthesized following the same procedure.

*Material characterization:* The crystal structure of samples was investigated by X-ray diffraction (XRD, Miniflex 600, Rigaku) with Cu-Kα radiation and Raman spectrometer (Horiba) with an excitation laser wavelength of 633nm. The internal structure of samples was determined using Transmission electron microscope (TEM, JEOL, JEM-2100F). The structural analysis and electrode morphology change were carried out using a field-emission scanning electron microscope (FE-SEM, JEOL) with an acceleration voltage of 15.0 kV. Pair distribution function (PDF) analysis was performed using synchrotron X-ray total scattering measurements with an incident X-ray energy of 75.051 keV (λ = 0.16520 Å). The surface area and pore size of samples were determined using N_2_ adsorption-desorption isotherms at 77 K (Autosorb-iQ3, Quantachrome) using the Brunauer-Emmett-Teller (BET) method. Chemical composition and depth profiling of the solid electrolyte interphase (SEI), along with the chemical bonding of the samples, were confirmed by X-ray photoelectron spectroscopy (XPS). The X-ray spot size of XPS was 400 μm, and the source gun type was Al K Alpha (2 keV). Gas evolution during the reduction was analyzed by gas chromatography (GC, 8860 GC system, Agilent). Temperature-programmed desorption (TPD) measurements were carried out on a catalyst analyzer (BELCAT ꠱, Microtrac). Atomic force microscopy (AFM, Bruker Dimension ICON) was used to analyze the electrode surface roughness.

*Electrochemical measurements:* Ge-based electrodes consist of active materials, PAA/CMC (1:1, w/w) as a binder, and Super-P as conducting agent with a weight ratio of 70:15:15. The mixed slurry was cast on Cu foil, dried in a vacuum oven at 150 ℃ for 6h. The prepared electrodes were cut into disks (diameters of 14mm, loading mass of 1.0-1.5 mg cm^-2^) and assembled into the CR2032 cells (Welcos) in an Argon-filled glove box using a Celgard separator, Li metal counter electrode (300 µm), and 75 μL of electrolytes dissolving 1.3 M LiPF_6_ in EC/DEC (3:7, v/v) with 10 wt % of fluoroethylene carbonate (FEC) additive. The galvanostatic battery tests on the anodes were conducted with the cut-off voltage of 0.005-1.5 V for the formation cycle at 0.05 C and 0.01-1.5 V for 0.2-5C, respectively, on a battery cycler (WBCS 3000, Wonatech) at 24 ℃. For Ge-based anodes | | LFP full cells, cut-off voltages are 2.5-3.9 V. Based on the cathode areal capacity of 1.7 mAh cm^-2^, the anode loading was adjusted accordingly, resulting in a full cell’s n/p ratio of ~1.1. The Cyclic voltammetry (CV) measurements were conducted at 0.1 mV s^-1^ and 0.01-1.5 V (VMP3, Biologic). The galvanostatic intermittent titration technique (GITT) of the assembled cells was carried out with 60 min of current pulse followed by relaxation for 90 min at 0.1C in the voltage range of 0.01-1.5 V. The chemical diffusion coefficient of the Li-ion was calculated by applying Fick’s second law of diffusion. After a series of assumptions and simplifications for small overpotentials with sufficiently small currents, the expression for $D_{{Li}^{+}}$ can be calculated as:

$$D_{{Li}^{+}}=\frac{4}{\pi\tau}\left( \frac{m_{B}V_{M}}{M_{B}S} \right)^{2}\left( \frac{\Delta E_{s}}{\Delta E_{t}} \right)^{2}$$

where $m_{B}$, $V_{M}$, $M_{B},$ and $S$ denote the mass, the molar volume, the molecular weight, and the active surface area of the anode electrode, respectively. $\Delta E_{s}$ is the difference between the steady-state potentials before and after a current pulse and thus indicates the change in the potential due to thermodynamics. $\Delta E_{t}$ is the magnitude of the potential changes (without the IR drop) during the current pulse. $\tau$ is the pulsed time. The EIS measurements were conducted between 100 kHz and 0.1 Hz with an amplitude of 10 mV. To formulate the compact electrode (Table S2), the proportion of active materials was increased to 85-92 wt% and the fabricated electrodes were further calendared with a rolll press machine at a press density of 15-30%.

*Pouch-type full cell fabrication:* To verify the feasibility of GN13 in Lithium-Ion Batteries, GN13/C composites were first prepared by blending GN13 with natural graphite at a weight ratio of 31:69. The resulting composite electrodes were designed to achieve a target specific capacity of ≈ 500 mAh g^-1^ in the blended electrodes. The GN13/C electrode consists of GN13/C composite, PAA as a binder, and Super-P as conducting agent with a weight ratio of 90:5:5. The GN13/C and LFP electrodes were prepared in dimensions of 3.4 × 4.3 cm^2^ and 3.3 × 4.2 cm^2^, respectively. Based on the cathode areal capacity of 1.7 mAh cm^-2^, the anode loading was adjusted accordingly, resulting in a full cell’s n/p ratio of ~1.1.


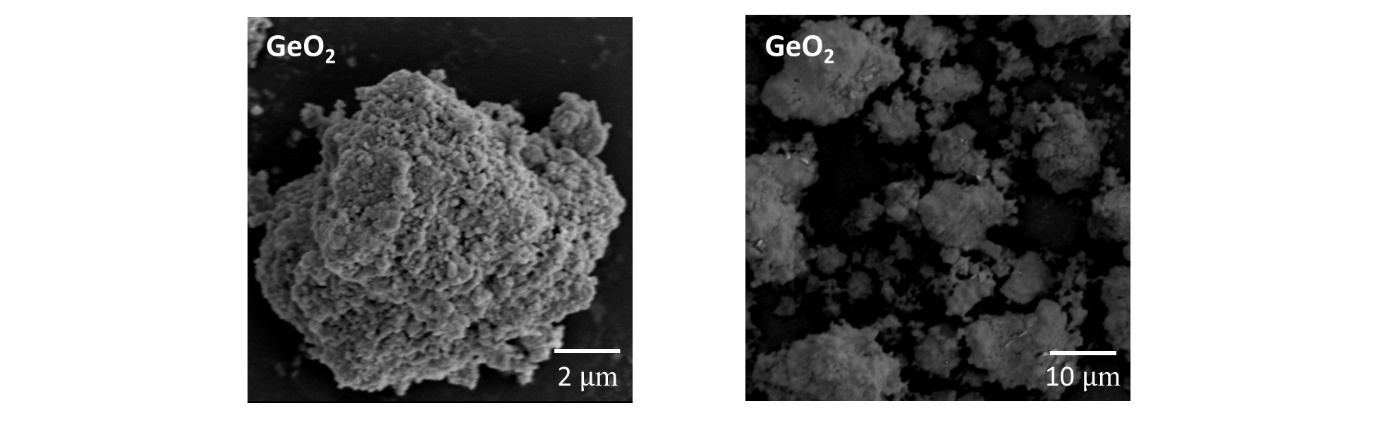


**Figure S1.** FE-SEM images of GeO_2_.


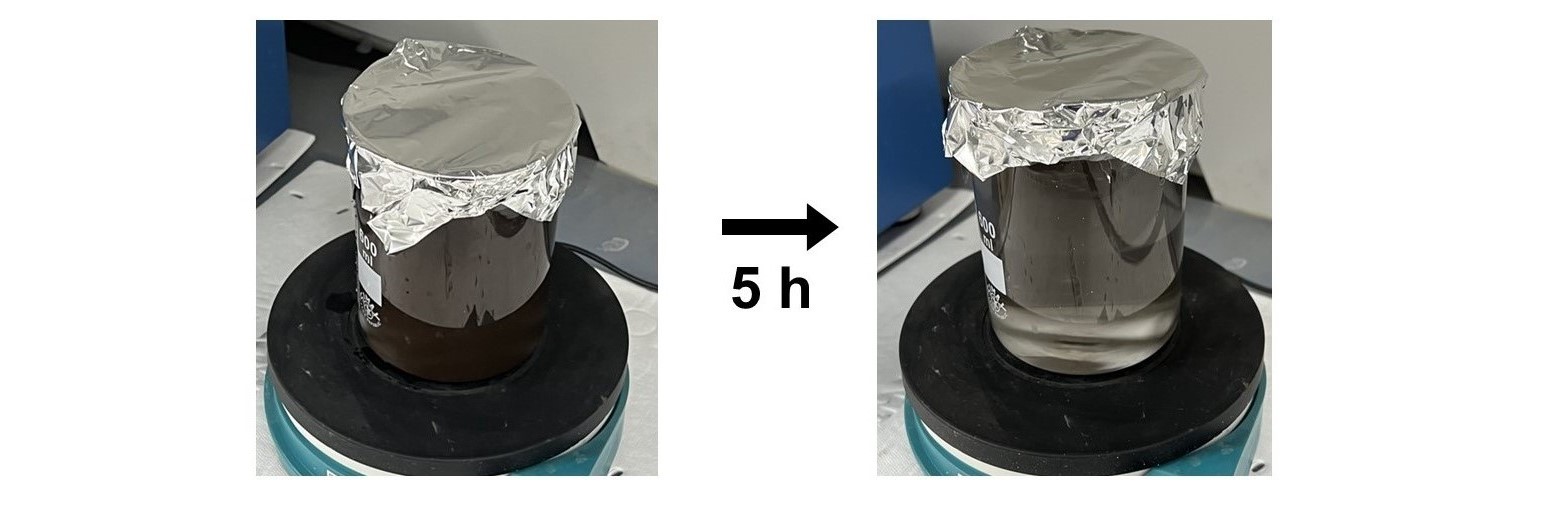


**Figure S2.** Optical images of GN14 during washing in deionized water.


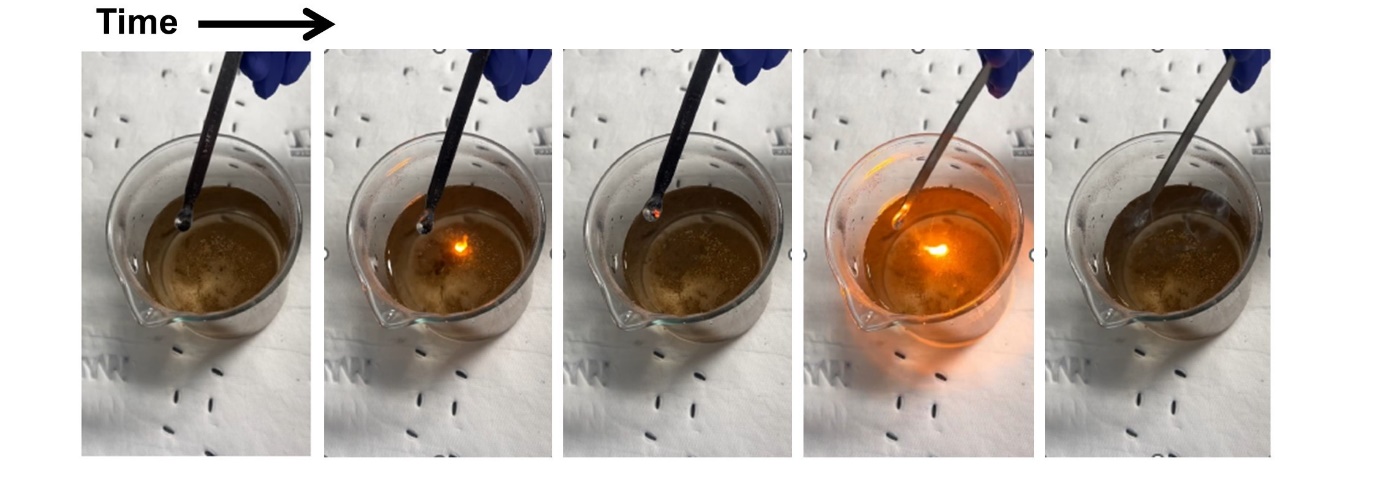


**Figure S3.** GN14 washing process after NaH-mediated reduction.


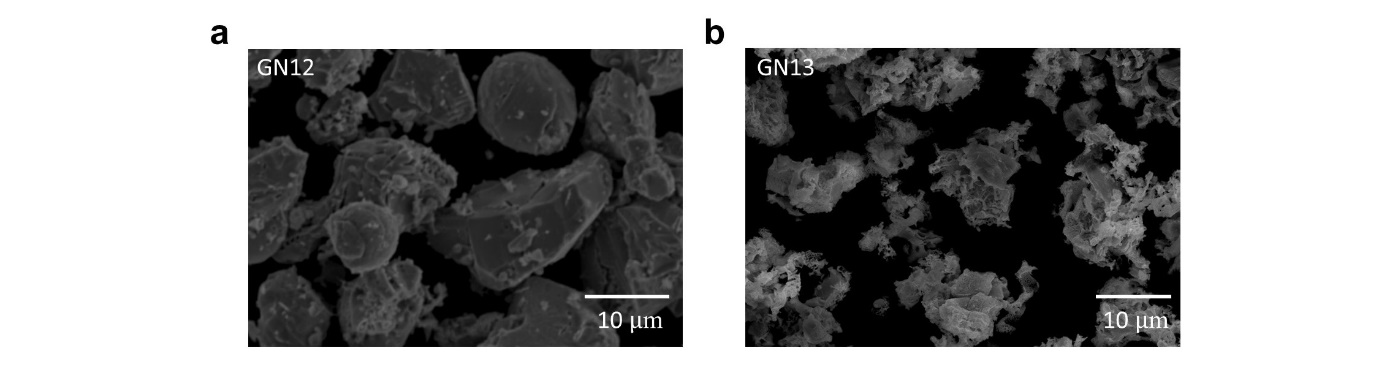


**Figure S4.** Size distribution. SEM images of (a) GN12 and (b) GN13.


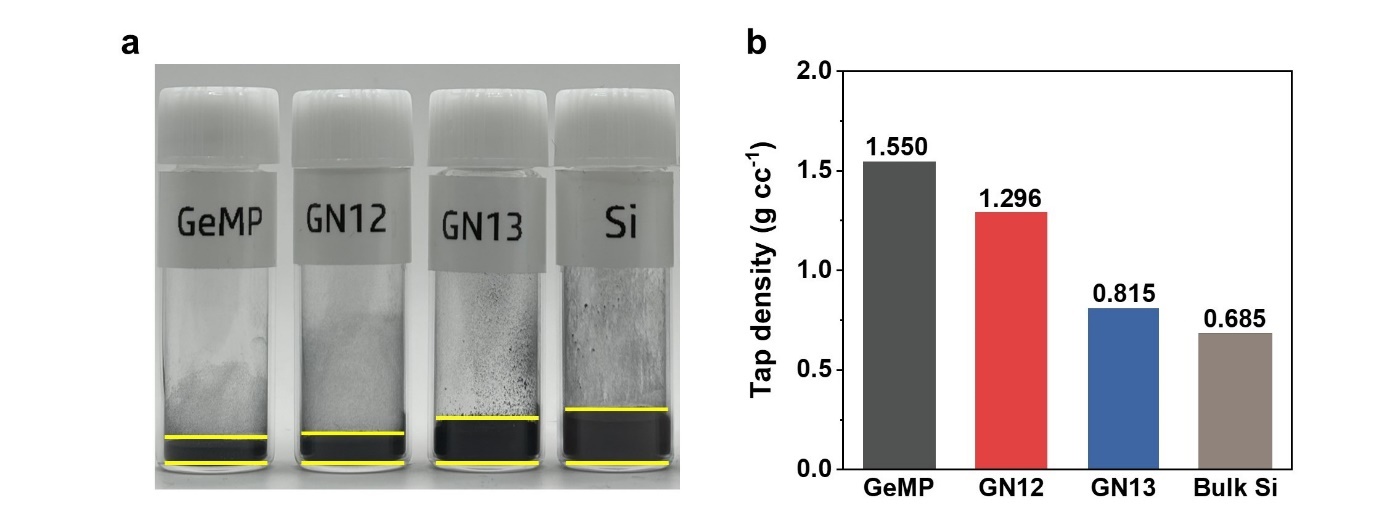


**Figure S5.** (a) Photographs of GeMP (10 μm), GN12, GN13, and bulk Si (5 μm) of tightly packed powder. (b) Tap density of GeMP, GN12, GN13 and bulk Si.


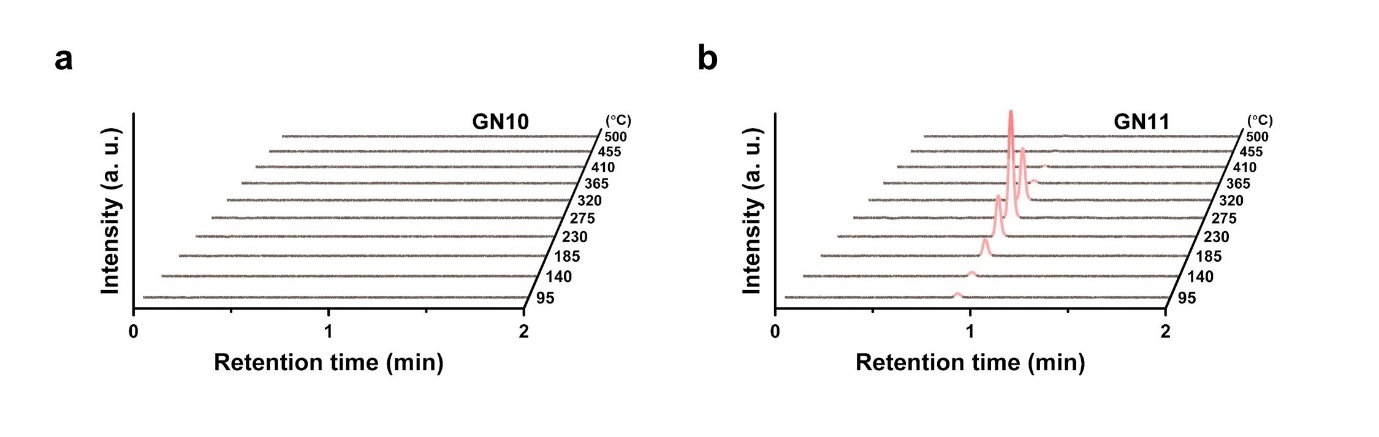


**Figure S6.** Temperature-dependent GC profiles of (a) GN10 and (b) GN11.

**
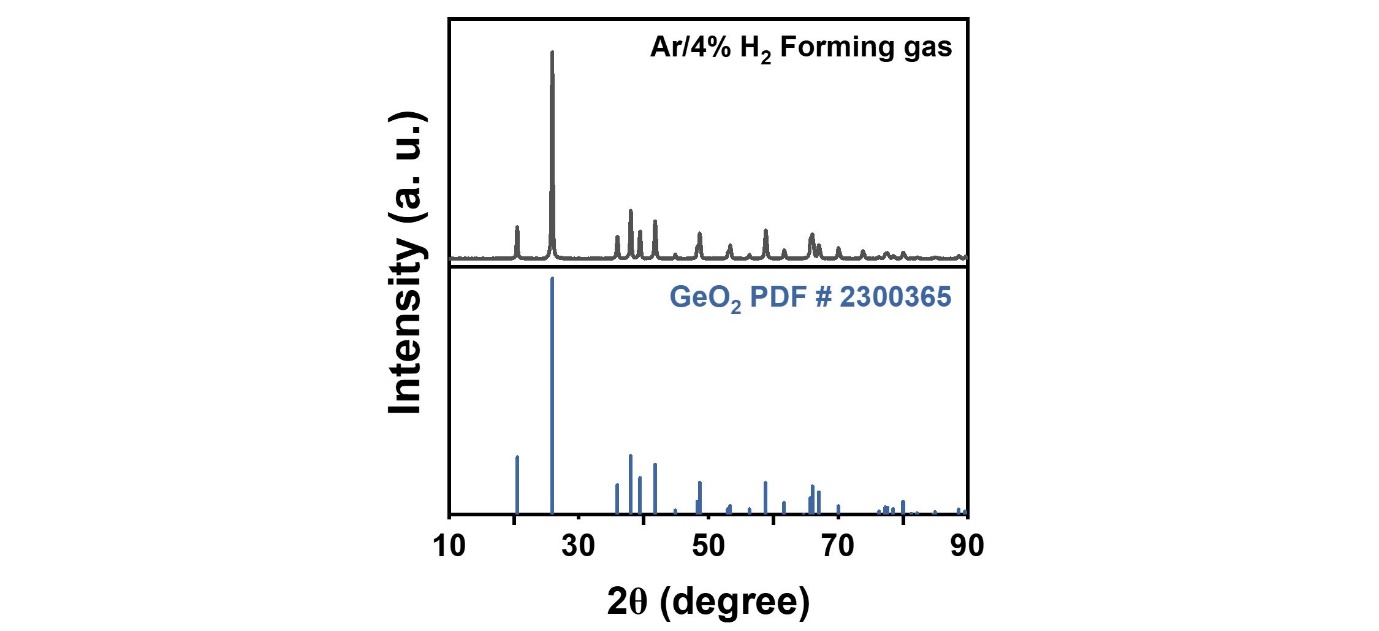
**

**Figure S7.** XRD patterns of GeO_2_ reduced in 4% H_2_ /Ar forming gas.

**
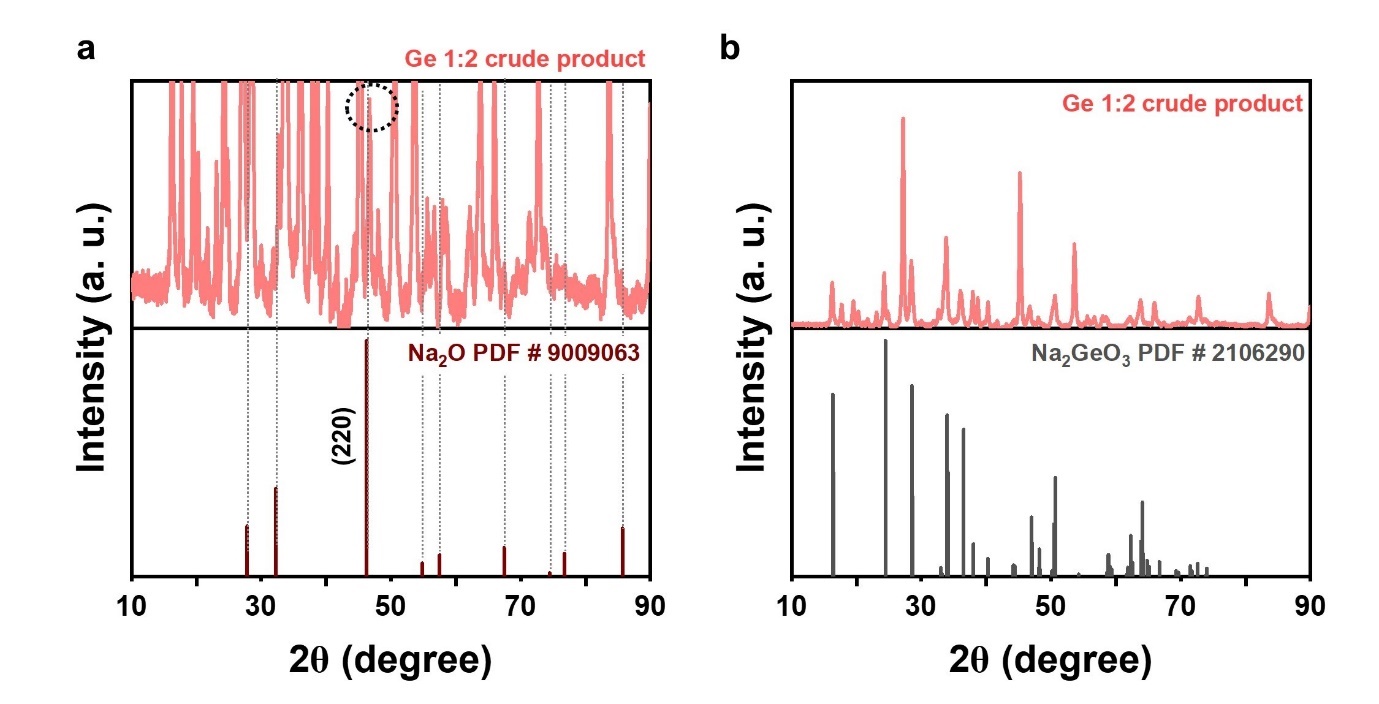
**

**Figure S8.** Enlarged XRD patterns of GN12 crude product.

**
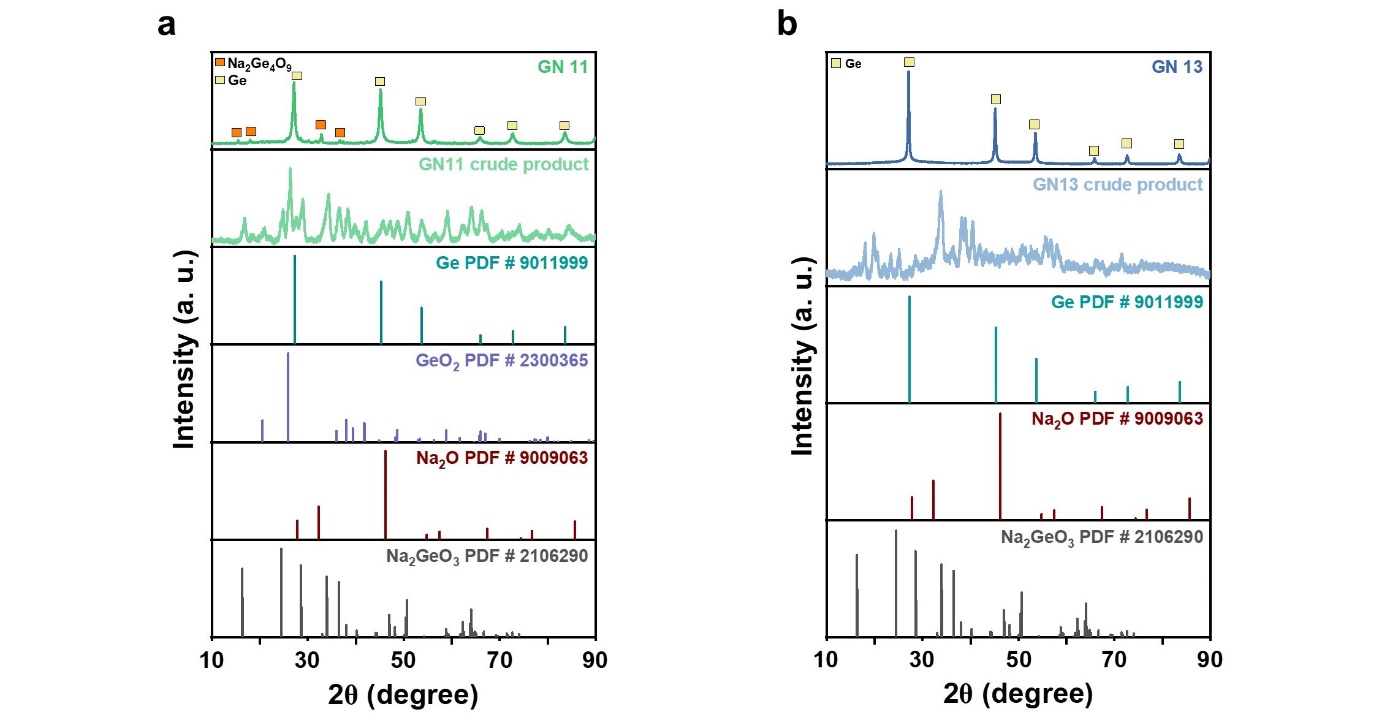
**

**Figure S9.** XRD patterns of (a) GN11 crude product and (b) GN13 crude product.


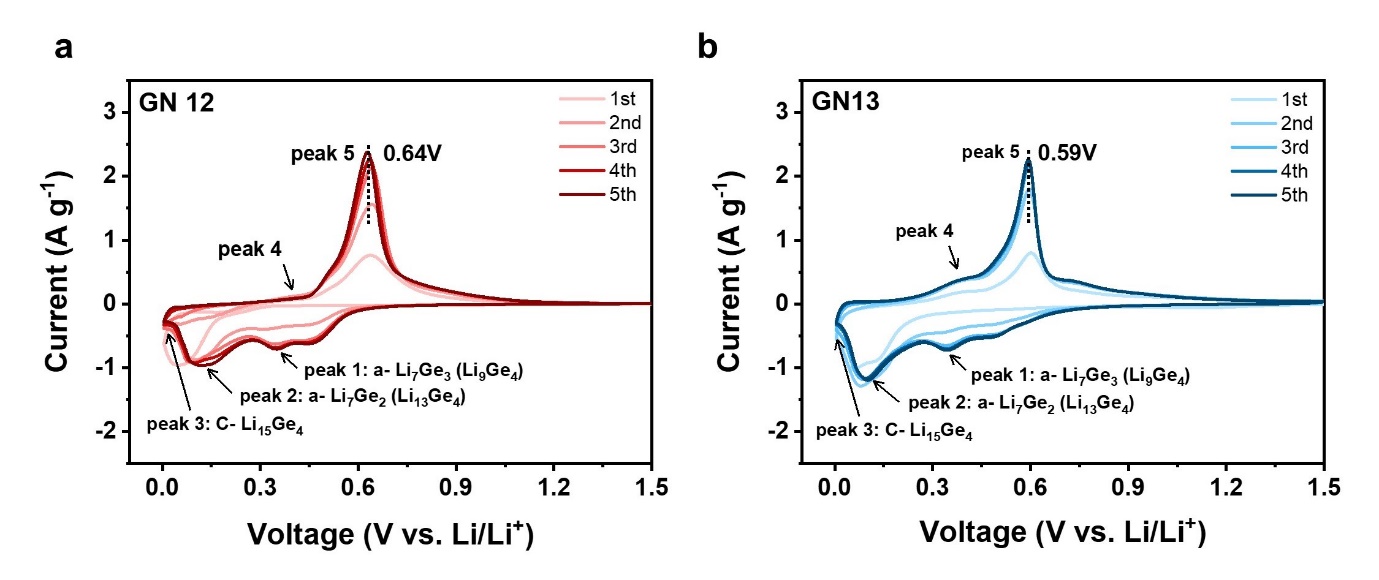


**Figure S10**. Cyclic voltammetry (CV) analysis of GN12 and GN13 electrodes with various cycle numbers. CV curves of (a) GN12 and (b) GN13 at a scan rate of 0.1 mV s^-1^.


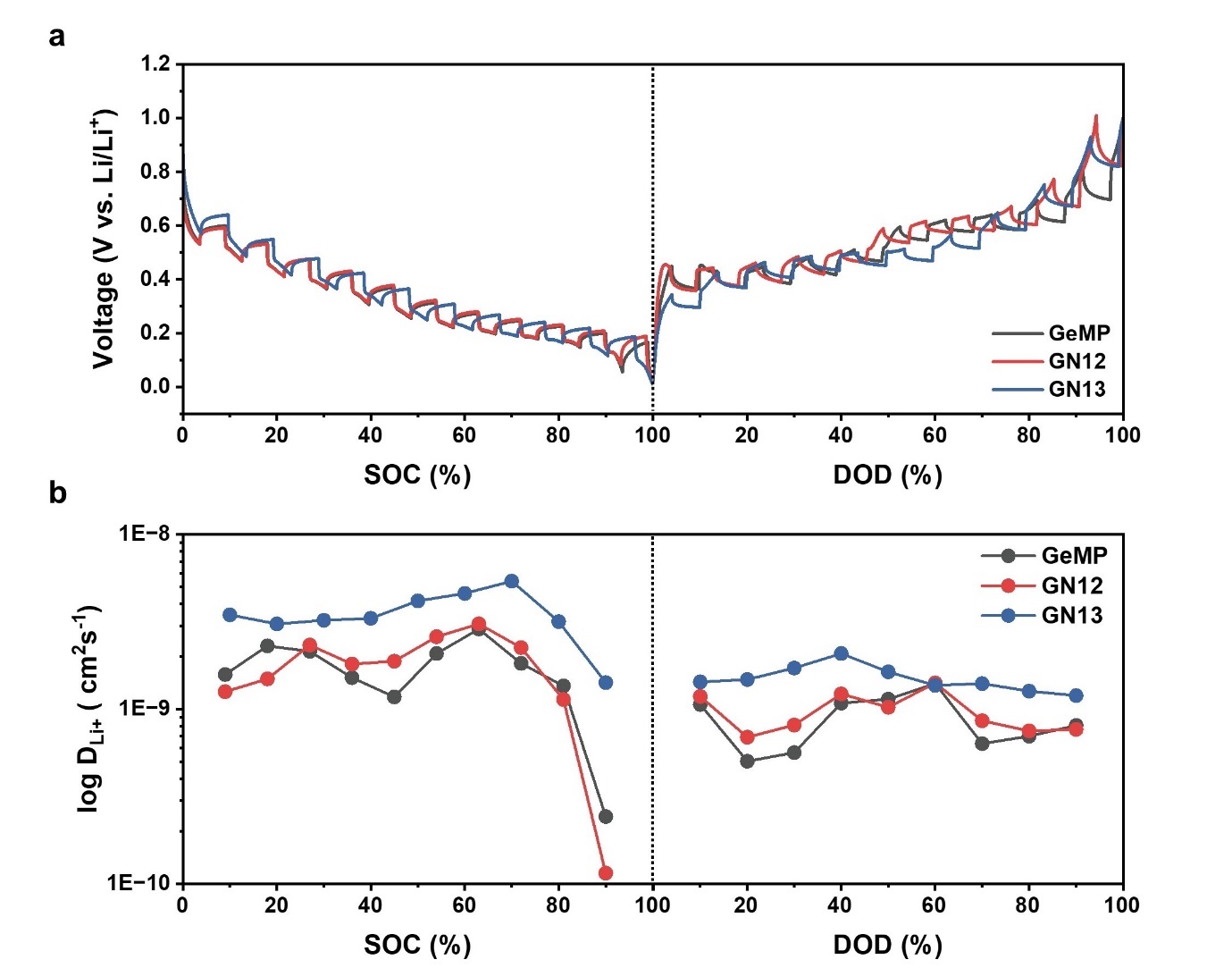
**Figure S11.** (a) GITT measurement results of GeMP, GN12, and GN13 electrodes. (b) Variation in Li-diffusion coefficient of GeMP, GN12, and GN13 electrodes versus the state of charge (SOC) measured by GITT.

**
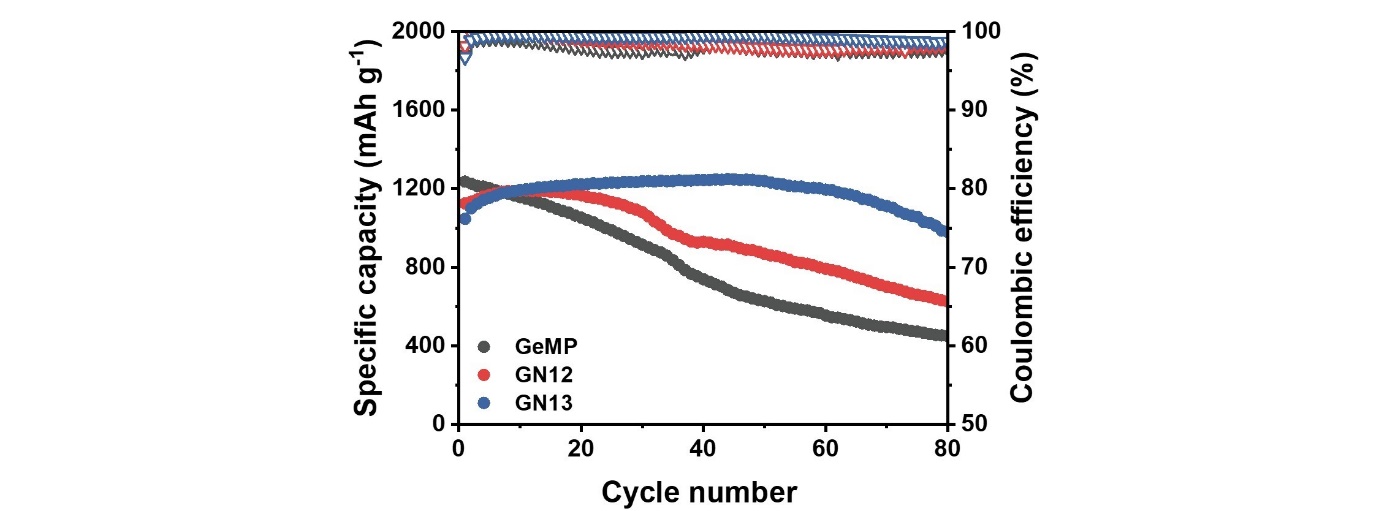
**

**Figure S12.** Electrochemical performance with half-cells of GeMP, GN12, and GN13 electrodes. Long-term cycling performance and corresponding columbic efficiencies (CEs) of the GeMP, GN12, and GN13 half-cells at 0.5C (1C = 1.2 A g^-1^).


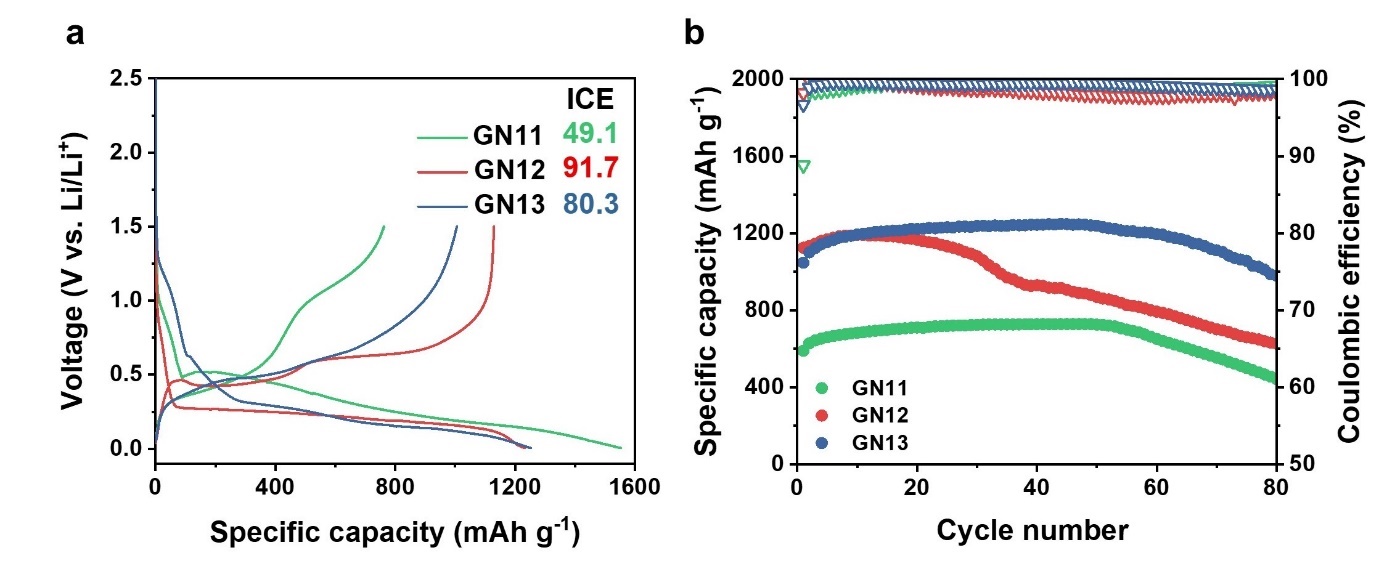


**Figure S13.** Electrochemical performance with half-cells of GN11 electrode. a) Galvanostatic charge-discharge profiles of GN11 electrodes. b) Long-term cycling performance and corresponding CEs of the GN11 electrodes at 0.5C (1C = 0.8 A g^-1^).


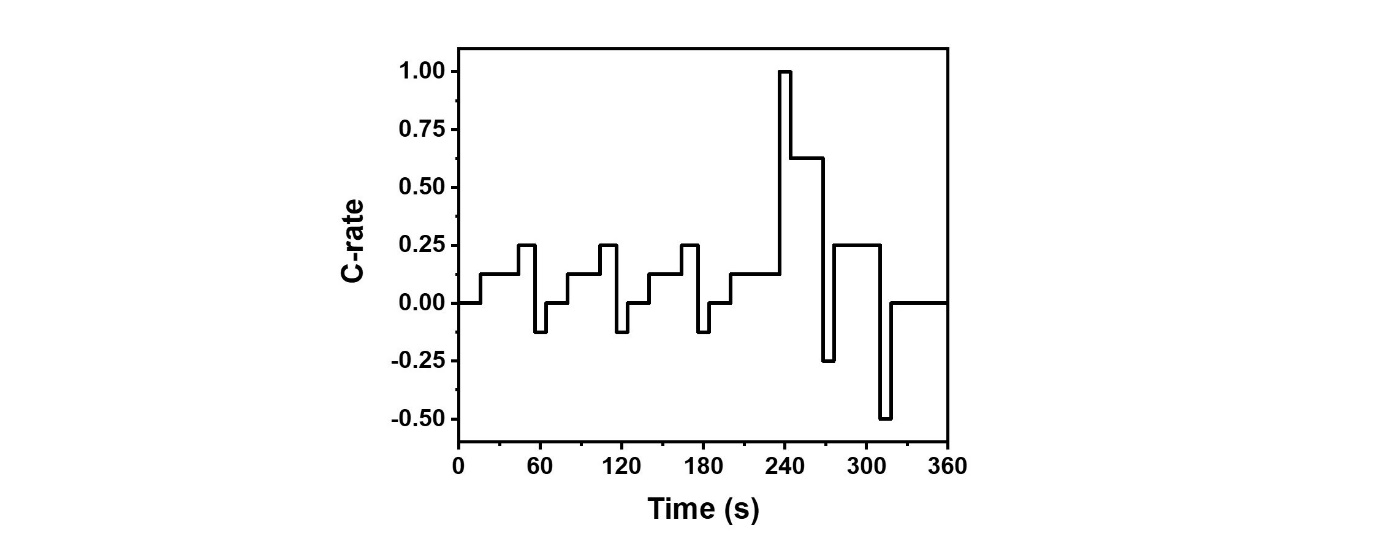


**Figure S14**. United States Advanced Battery Consortium (USABC) dynamic stress test (DST) protocol shown in the form of a C-rate versus time plot.

**
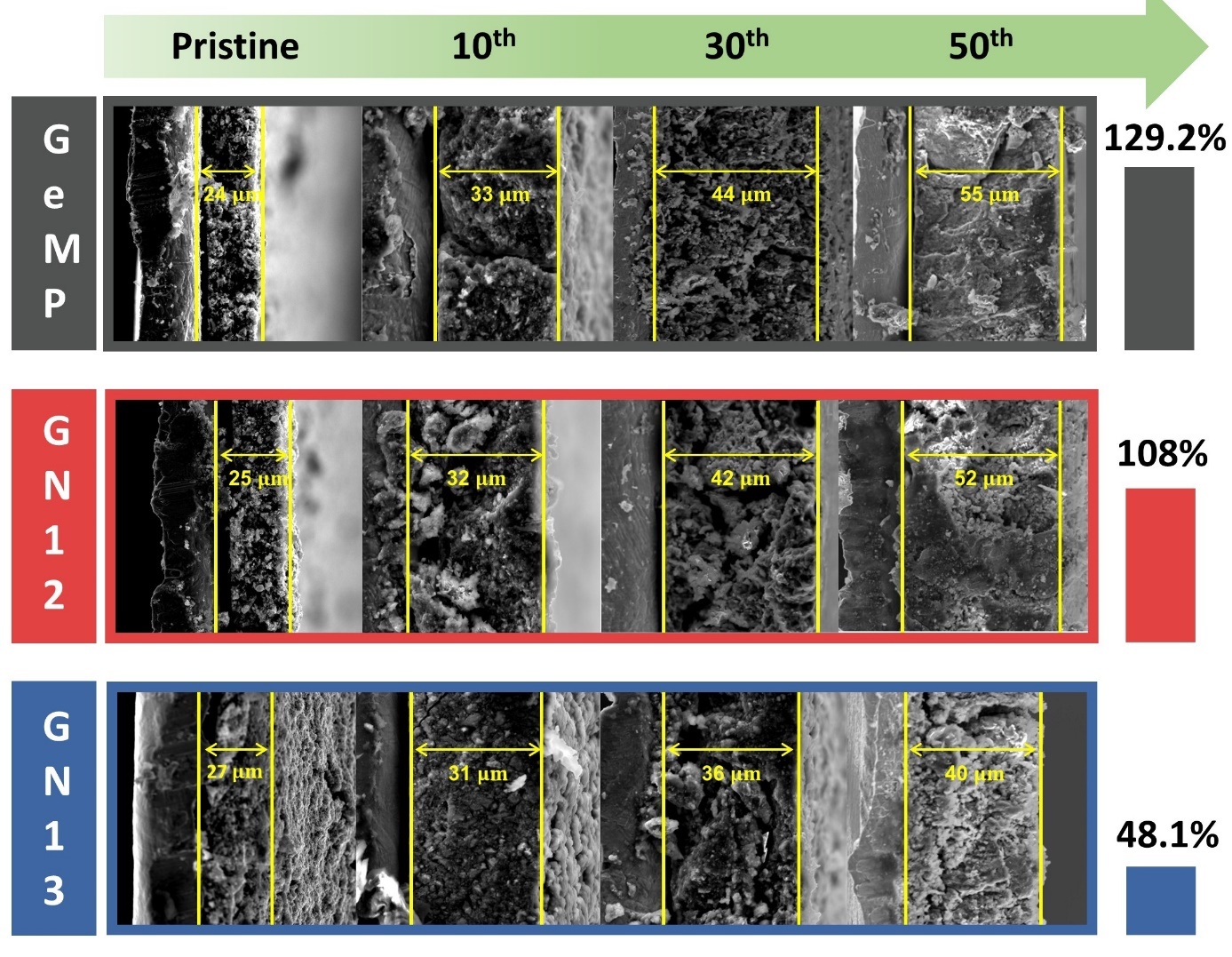
**

**Figure S15.** Electrode swelling test of GeMP, GN12, and GN13 electrodes over cycling and comparison of volume expansion.


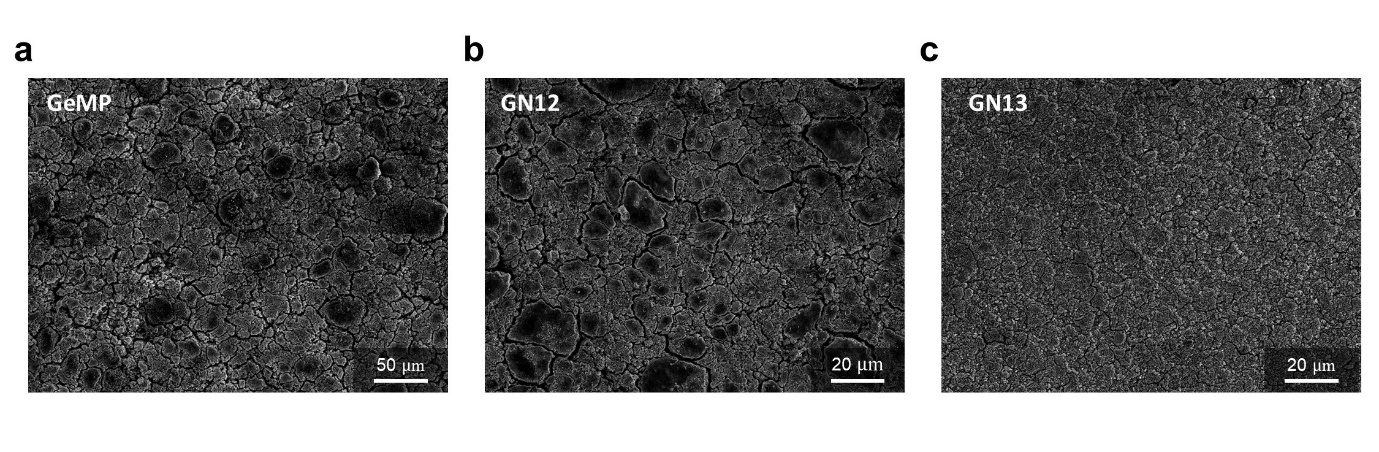


**Figure S16.** Top-view FE-SEM images of (a) GeMP, (b) GN12, and (c) GN13 electrodes after 50 cycles.


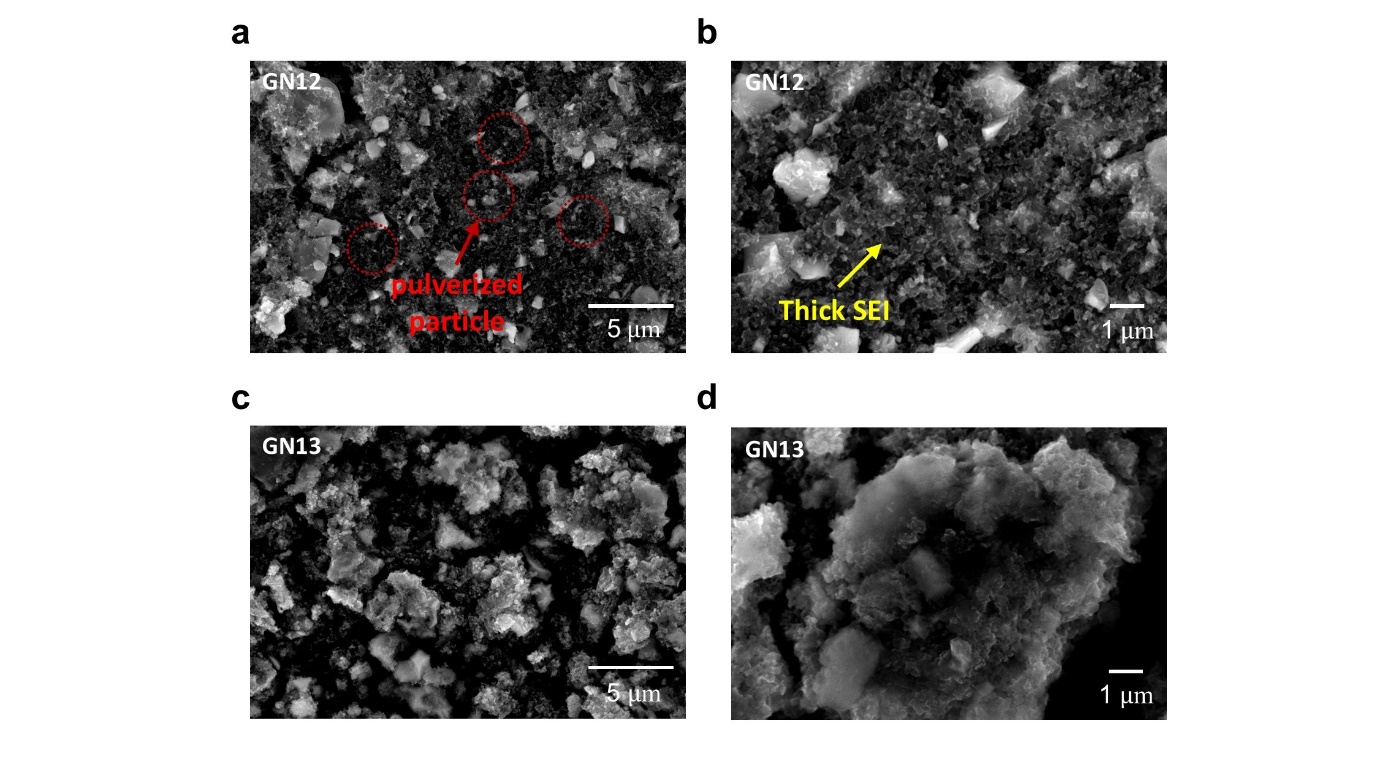


**Figure S17.** FE-SEM images of (a, c) GN12 and (b, d) GN13 particles after 30 cycles.


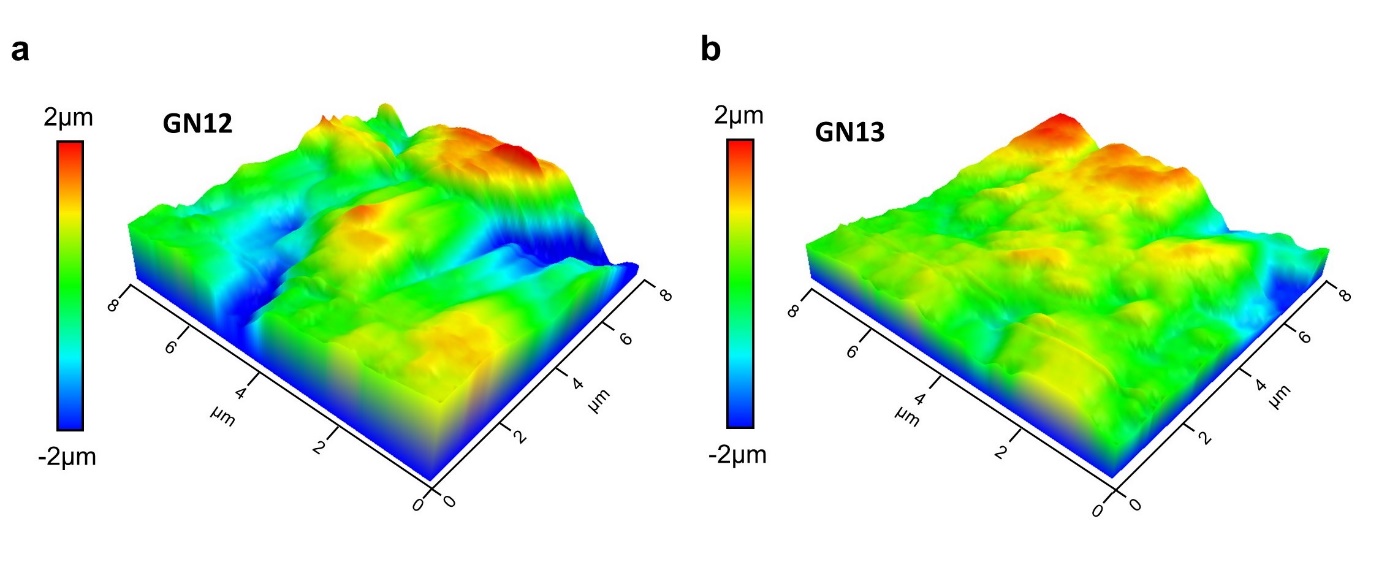


**Figure S18.** Atomic forcoscopy (AFM) surface morphology of (a) GN12 and (b) GN13 electrodes after 50 cycles.


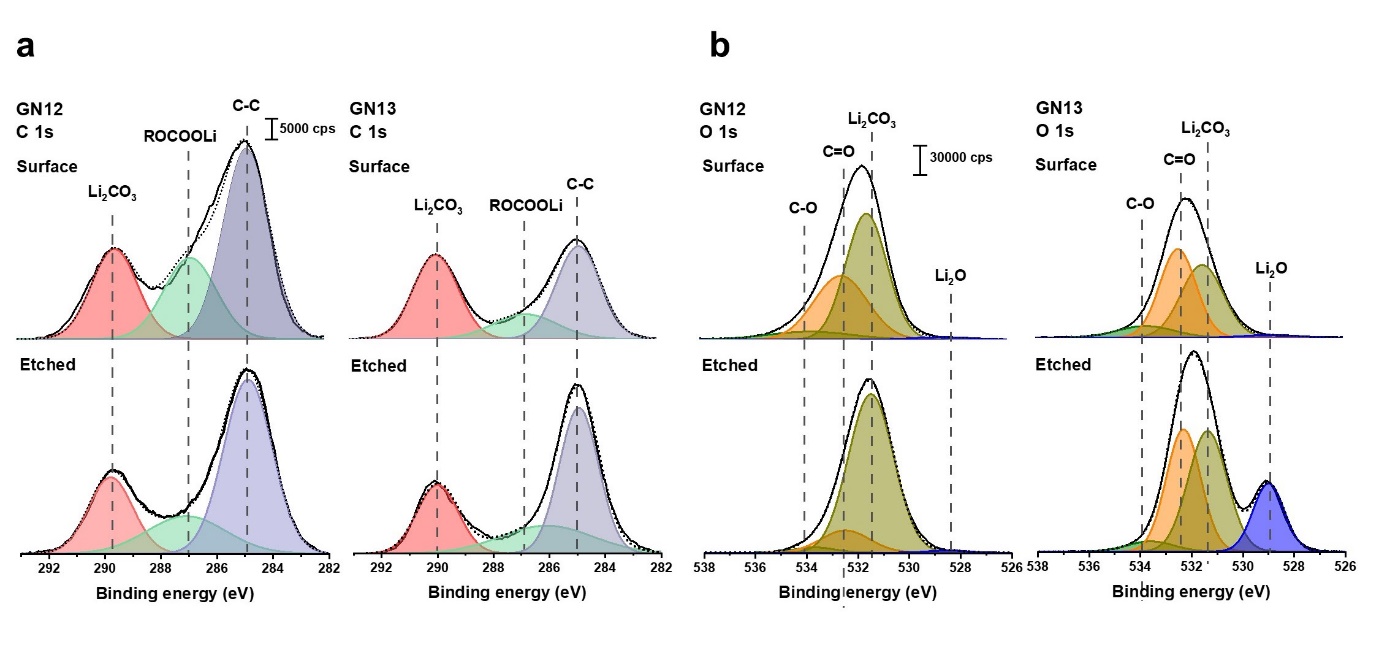


**Figure S19.** In-depth XPS spectra (C 1s and O 1s) of the (a) GN12 and (b) GN13 electrodes after 50 cycles.


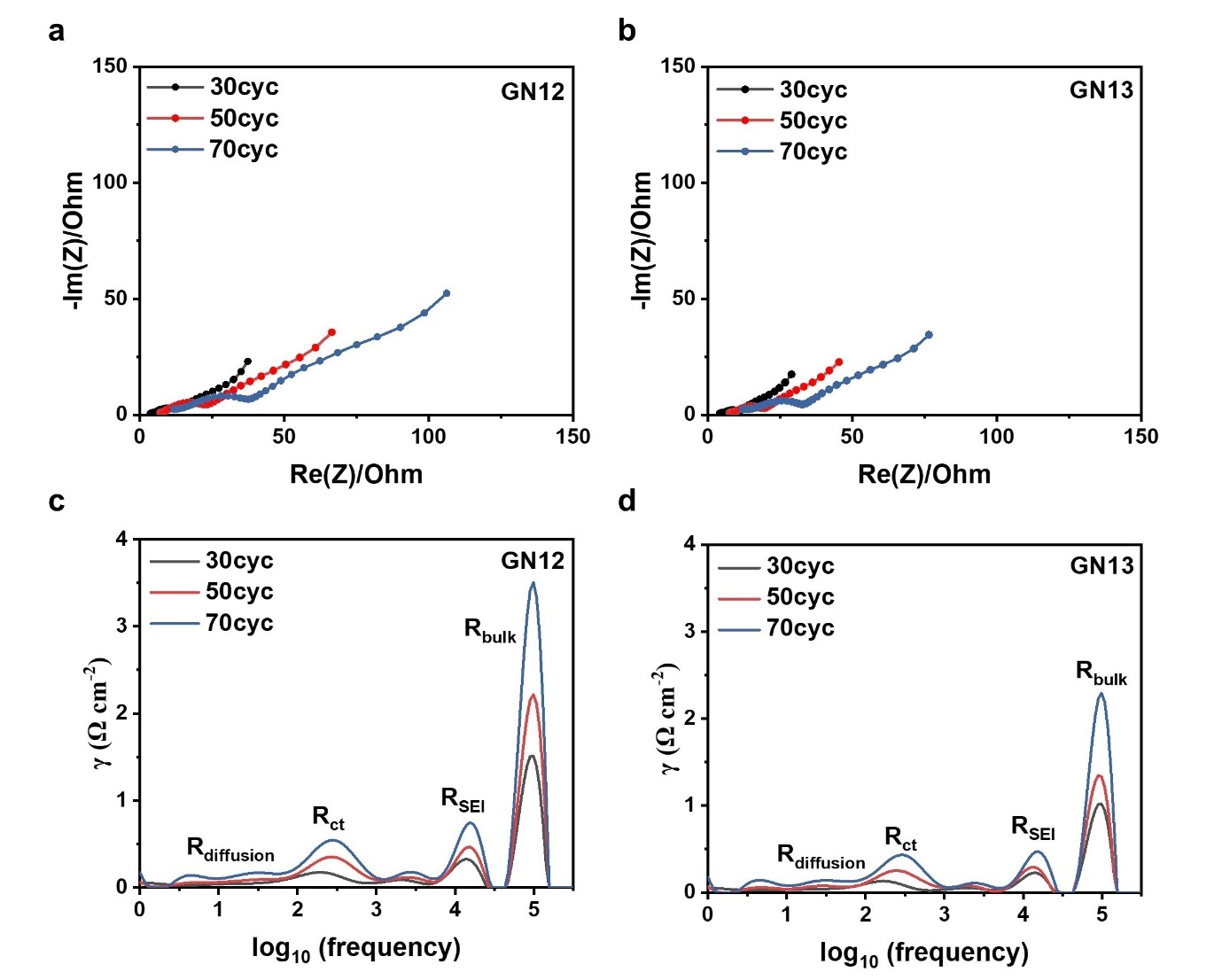


**Figure S20**. EIS measurements of GN12 and GN13. Nyquist plots for the (a) GN12 and (b) GN13 electrodes over cycling. DRT analysis from EIS data for (c) GN12 and (d) GN13 electrodes over cycling.


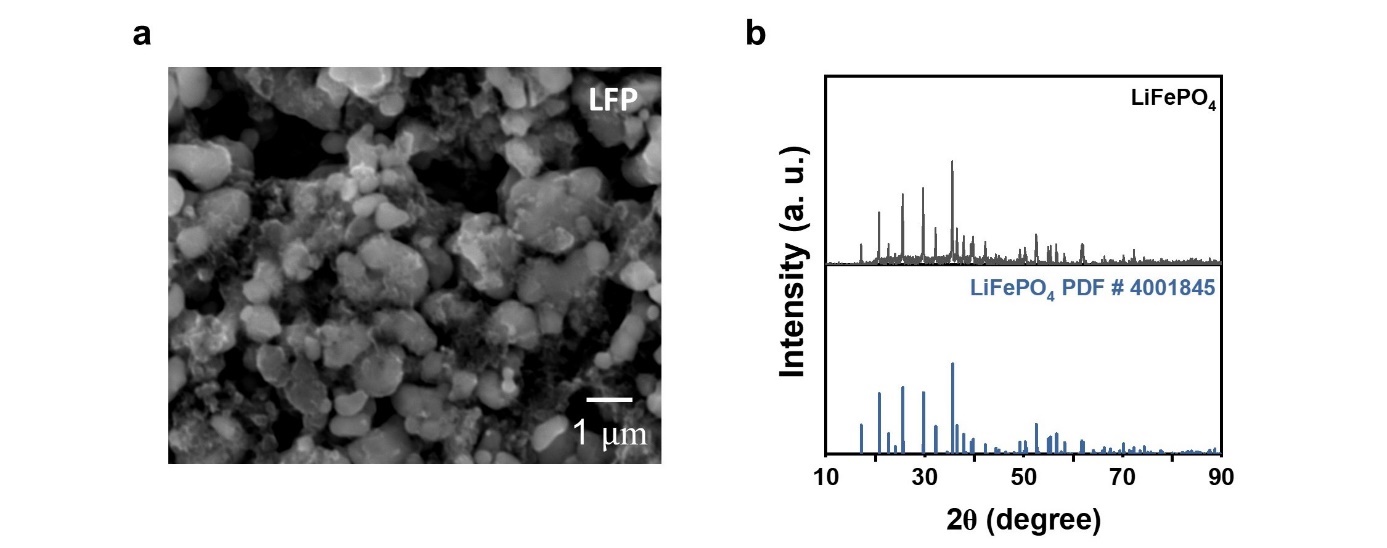


**Figure S21.** (a) FE-SEM image and (b) XRD pattern of commercial LFP electrode**.**


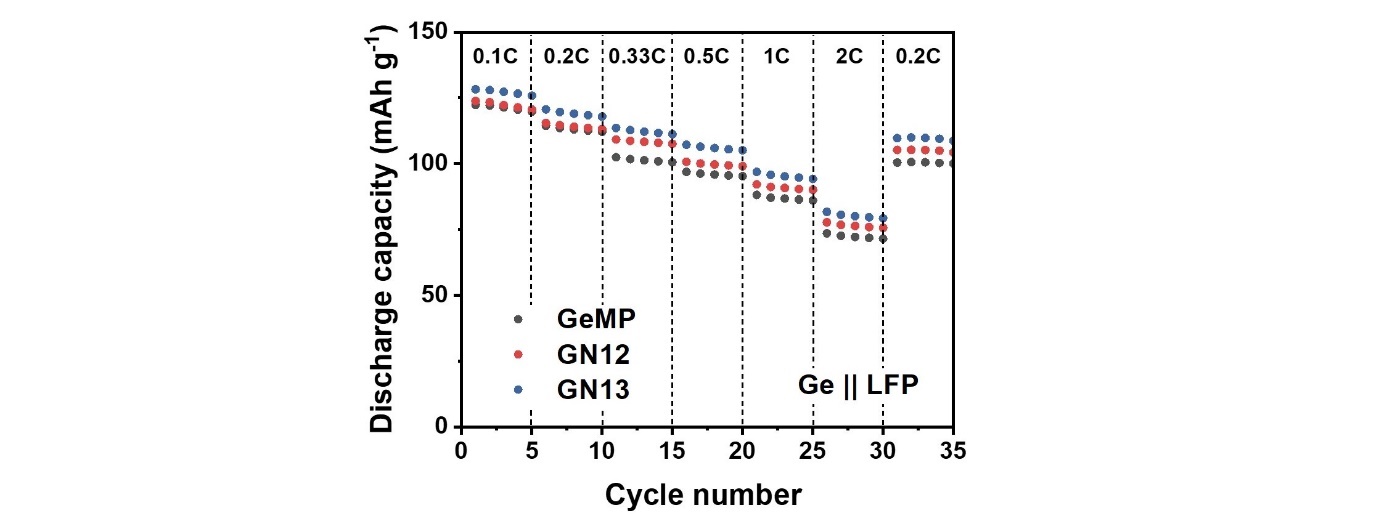


**Figure S22.** Rate capability of GeMP || LFP, GN12 || LFP, and GN13 || LFP full cells.


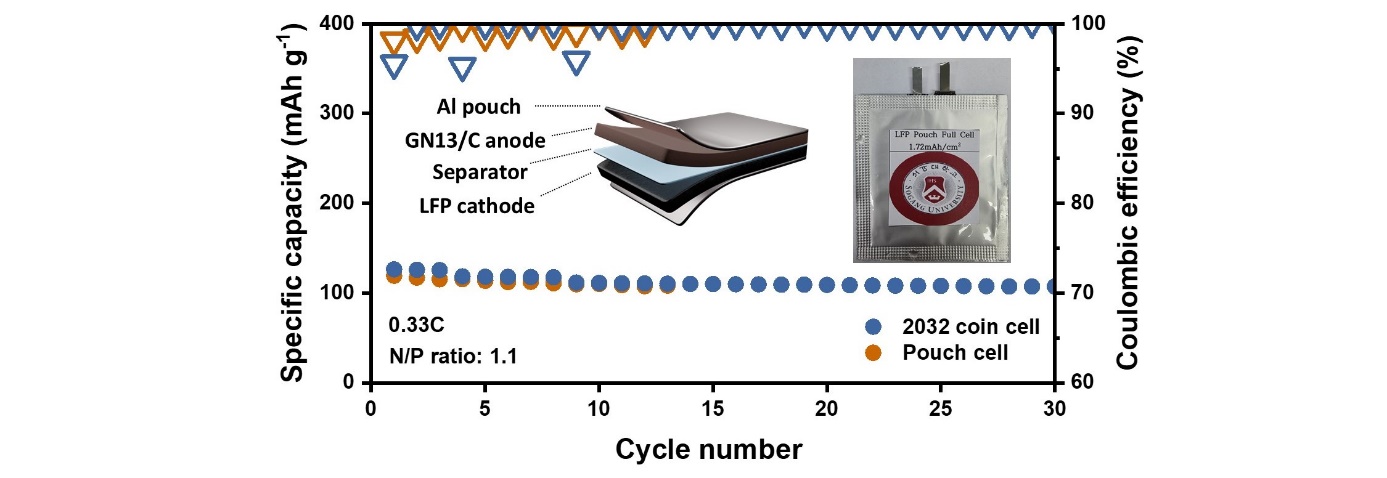


**Figure S23.** Cycle performance of pouch-type GN13/C || LFP full cell.

**Table S1.** Cycling performance comparison of Ge-based half-cells with various synthesized Ge particle sizes.

| Ref. | Active material | Size (μm) | Synthesis method | Structure | Active material mass loading (mg cm^-2^) | Electrode packing density  (g cc^-1^) | Half-cell | |
| --- | --- | --- | --- | --- | --- | --- | --- | --- |
|  |  |  |  |  |  |  | ICE (%) | Cycle retention  Current density |
| **This work** | **GN13** | **5 - 8** | **Hydride-mediated reduction** | **Nonporous** | **1.5** | **0.56** | **80.3** | **83.0%@160th**  **(0.24 A g^-1^)** |
|  | **GN12** | **7 - 10** |  | **Mesoporous** | **1.5** | **0.60** | **91.7** | **76.5%@80th**  **(0.24 A g^-1^)** |
| S1^[1]^ | Z-Ge | 15 | Magnesiothermic reduction | Mesoporous | 0.8 - 1.5 |  | 65.5 | 97.3%@150th (0.2C) |
| S2^[2]^ | NG-21 | 0.20 | Zincothermal reduction | Nanoporous | 1.2 - 2.1 | 0.71 | 85.3 | 100.0%@100th (0.2C) |
| S3^[3]^ | 3D-pGe | 1 - 2 | Thermal deformation  and hydrogen reduction | 3D porous | 1.2 - 1.4 | 1.01 | 92.3 | 83%@250th  (1C) |
| S4^[4]^ | Ge NTs | 0.2 - 0.25 | Kirkendall effect | Nanotube |  | 1.20 | 76 | 92%@50th  (0.2C) |
| S5^[5]^ | Ge NWs | 0.06 | Liquid-injection chemical vapor deposition (LICVD) | Nanowire | 0.6 |  | 42 | 79.5%@100th  (0.2C) |
| S6^[6]^ | Ge-p-40 | 0.05 - 0.1 | Molten-salt electrolysis | Nanowire | 2.0 |  | 81.4 | 81.7%@300th  (0.3 A g^-1^) |
| S7^[7]^ | Ge NW | 0.05 - 0.1 | Solution-liquid-  Solid (SLS) growth | Nanowire |  |  | 51.2 | 34.3%@50th  (0.1C) |
| S8^[8]^ | NPGeNF | 0.2 | Electrospinning and zincothermic reduction reaction (ZRR) | Nanoporous  and  nanofiber | 1.0 | 0.72 | 81.8 | 80.4%@500th (3.0C) |

**Table S2**. Electrode and materials porosity and comparison of volumetric capacity of Ge-based electrodes before and after reformulation.

| Samples | P_anode_^a^ / % | *r*P_anode_^b^ / % | P_intra_ / % | P_inter_ / % | *r*P_inter_ / % | C_volumetric_^c^ / mAh cm^-3^ | |
| --- | --- | --- | --- | --- | --- | --- | --- |
|  |  |  |  |  |  | Before | After |
| GeMP | 81.1 | **53.6** | 0 | 81.1 | 53.6 | 837 | **1058** |
| GN12 | 81.8 | **58.6** | 16.4 | 65.4 | 42.2 | 677 | **940** |
| GN13 | 83.0 | **68.7** | 47.4 | 35.6 | 21.3 | 588 | **793** |

^a^P_anode_ = P_intra_ + P_inter_, ^b^*r*P_anode_ = P_intra_ + *r*P_inter_, ^c^Capacities are calculated based on the initial reversible specific capacity and formulated and calendared electrode thickness

**Supporting References**

[1] N. Lin, T. Q. Li, Y. Han, Q. L. Zhang, T. J. Xu, Y. T. Qian, *ACS Appl. Mater. Interfaces* 2018, **10**, 8399.

[2] D. Kwon, J. Ryu, M. Shin, G. Song, D. Hong, K. S. Kim, S. Park, *J. Power Sources* 2018, **374**, 217.

[3] T. Yoon, G. Song, A. M. Harzandi, M. Ha, S. Choi, S. Shadman, J. Ryu, T. Bok, S. Park, K. S. Kim, *J. Mater. Chem. A* 2018, **6**, 15961.

[4] M. H. Park, Y. Cho, K. Kim, J. Kim, M. L. Liu, J. Cho, *Angew Chem Int Edit* 2011, **50**, 9647.

[5] D. McNulty, S. Biswas, S. Garvey, C. O'Dwyer, J. D. Holmes, *ACS Appl. Energy Mater.* 2020, **3**, 11811.

[6] H. Liu, T. H. Wu, L. Q. Zhang, X. Wang, H. F. Li, S. Q. Liu, Q. Zhang, X. Zhang, H. J. Yu, *ACS Nano* 2022, **16**, 14402.

[7] L. P. Tan, Z. Y. Lu, H. T. Tan, J. X. Zhu, X. H. Rui, Q. Y. Yan, H. H. Hng, *J. Power Sources* 2012, **206**, 253.

[8] C. Kim, G. Song, L. L. Luo, J. Y. Cheong, S. H. Cho, D. Kwon, S. Choi, J. W. Jung, C. M. Wang, I. D. Kim, S. Park, *ACS Nano* 2018, **12**, 8169.
